# Alzheimer’s disease polygenic risk, *APOE* ε4 dose, and accelerometer-derived circadian rest–activity rhythms in relation to incident dementia and Alzheimer’s disease in UK Biobank: a prospective cohort study

**DOI:** 10.1186/s13195-026-02088-3

**Published:** 2026-06-01

**Authors:** Simin Yang, Xiong Xiao, Yuqian Yang, Yinqian Cai, Yuxi Zou, Yanping Tang, Huihan Ma, Zelin Lai

**Affiliations:** 1 Neurosurgery Center, National Key Clinical Specialty, Engineering Research Center of Diagnostic and Therapeutic Technology and Devices for Cerebrovascular Diseases in Ministry of Education, Guangdong Provincial Key Laboratory on Brain Function Repair and Regeneration, Zhujiang Hospital Institute for Brain Science and Intelligence, Zhujiang Hospital, Southern Medical University, Guangzhou, 510282 China; 2 Ultrasound Medical Center, Zhujiang Hospital, Southern Medical University, Guangzhou, 510282 China; 3 Guangdong Clinical Research Academy of Chinese Medicine, The First Affiliated Hospital of Guangzhou University of Chinese Medicine, Guangzhou, 510405 China

**Keywords:** Alzheimer’s disease, Polygenic risk score, *APOE*, Accelerometry, Circadian rhythm, Incident dementia

## Abstract

**Background:**

Circadian rest–activity rhythm disruption and sleep–wake fragmentation are common in Alzheimer’s disease (AD) and may precede clinical diagnosis. Wrist-worn accelerometry enables objective, scalable circadian phenotyping in population cohorts. We examined whether AD polygenic risk and *APOE* ε4 dose are reflected in accelerometry-derived circadian phenotypes and whether these phenotypes predict subsequent incident dementia/AD.

**Methods:**

We analyzed 94,241 UK Biobank participants with 7-day wrist accelerometry and genetic data. Circadian phenotypes included relative amplitude (RA), L5 and M10 start hour, interdaily stability (IS), and intradaily variability (IV). Genetic exposures were a standardized AD polygenic risk score (AD-PRS) corresponding to the dataset-provided UK Biobank AD PRS variable standardized within the analytic sample, and APOE ε4 dose. Cross-sectional associations were tested with linear regression. Incident dementia/AD were modeled with Cox proportional hazards models with time zero defined as the accelerometry end date. Prespecified sensitivity analyses included lagged exclusions, competing risk of death, selection into accelerometry (inverse probability weighting), additional adjustment for overall activity, strict versus broad AD definitions, and RA × genetic-risk interaction terms in incident models.

**Results:**

AD-PRS showed no robust association with RA or phase timing (fully adjusted standardized beta = 0.0028; *p* = 0.358). APOE ε4 dose was associated with earlier M10 timing (standardized beta = -0.0074; *p* = 0.032). AD-PRS showed a nominal association with IS (standardized beta = 0.0073; *p* = 0.018) that did not survive multiple-testing correction. In incident analyses (*n* = 80,378; 949 dementia and 371 AD events), AD-PRS and APOE ε4 dose strongly predicted incident dementia and incident AD. Each 1-SD higher RA was associated with lower risk of incident dementia (HR = 0.80 per SD; *p* = 4.99 × 10^–19^) and incident AD (HR = 0.90; *p* = 0.031), meaning that lower RA corresponded to higher risk. The RA–dementia association remained stable across sensitivity analyses, whereas the RA–AD association attenuated after additional adjustment for overall activity. We found no evidence that RA associations differed by AD-PRS or APOE ε4 dose in incident interaction analyses (all interaction *p* values ≥ 0.362).

**Conclusions:**

In this population cohort, AD polygenic risk showed minimal cross-sectional association with circadian amplitude or phase, whereas *APOE* ε4 dose was linked to slightly earlier peak daytime activity timing. Lower circadian amplitude (RA) consistently predicted higher subsequent dementia risk across multiple bias-oriented sensitivity analyses. For incident AD, the RA association was weaker after additional control for overall activity, supporting cautious interpretation of RA as a robust behavioral risk marker for dementia rather than an AD-specific genetic interaction signal.

**Supplementary Information:**

The online version contains supplementary material available at https://doi.org/10.1186/s13195-026-02088-3.

## Background

Circadian system dysregulation and sleep–wake fragmentation are frequently observed in Alzheimer's disease (AD) and related dementias, can appear in preclinical stages, and are also associated with increased subsequent dementia risk in prospective studies and meta-analyses [1–3]. However, disentangling prodromal changes from potentially modifiable risk markers remains challenging in conventional clinic-based studies.

Mechanistically, circadian clocks coordinate sleep–wake regulation, neuronal excitability, metabolic homeostasis, and inflammatory tone, and are increasingly implicated in pathways relevant to AD pathogenesis [1, 2]. From a prevention and risk-stratification perspective, a central challenge is to determine which aspects of rest–activity disruption reflect prodromal neurodegeneration versus potentially modifiable behavioral signatures that are detectable years before diagnosis.

Objective quantification of rest-activity rhythms in large populations is now feasible via wrist-worn accelerometry, enabling standardized derivation of rhythm amplitude, phase timing, and nonparametric indices of stability and fragmentation [4–6]. These measures provide scalable digital phenotypes, but metrics such as relative amplitude (RA) are also correlated with overall activity and general health, raising questions about specificity to circadian regulation versus behavioral activity patterns.

Large population cohorts with linked genetics and long-term follow-up provide an opportunity to test whether digital circadian phenotypes add information beyond established risk factors, while explicitly addressing interpretability issues common to observational wearable studies, including early-event/prodromal bias, selection into accelerometry subcohorts, and competing risk of death.

Genetic susceptibility to AD includes both major-effect loci and broader polygenic susceptibility. *APOE* ε4 dose is the strongest common genetic risk factor for late-onset AD [7], whereas polygenic risk score (PRS) variables summarize distributed genome-wide risk captured by upstream PRS resources rather than the total latent genetic contribution to disease risk [8–10]. Prior UK Biobank studies have linked accelerometer-derived rhythm measures and physical activity to dementia risk, but have less clearly tested whether AD genetic risk is mirrored in these behavioral circadian phenotypes or whether rhythm-related associations differ across genetic-risk strata.

Using UK Biobank, we therefore aimed to (i) quantify cross-sectional associations of AD-PRS and *APOE* ε4 dose with accelerometry-derived circadian phenotypes (RA, L5/M10 start hour, IS, IV); (ii) evaluate whether genetic and circadian phenotypes predict incident dementia and incident AD after accelerometry; and (iii) formally test whether the RA association differed by AD-PRS or *APOE* ε4 dose in incident models.

## Methods

### Study design and reporting

We conducted an observational analysis using UK Biobank data accessed on the Research Analysis Platform (RAP) [11, 12]. Reporting follows STROBE guidelines for observational research [13].

We used an AI-assisted language tool to improve grammar and clarity during manuscript preparation; all authors reviewed and edited the content and take full responsibility for the final version.

Time zero (t0) for time-to-event analyses was defined as the accelerometry end date, ensuring that circadian phenotypes were measured prior to the start of prospective follow-up. We treated accelerometry-derived circadian metrics as baseline exposures for incident dementia/AD analyses, and we used the same covariate definitions across cross-sectional and incident models to maximize comparability.

### Participants and cohort assembly

**Fig. 1:**
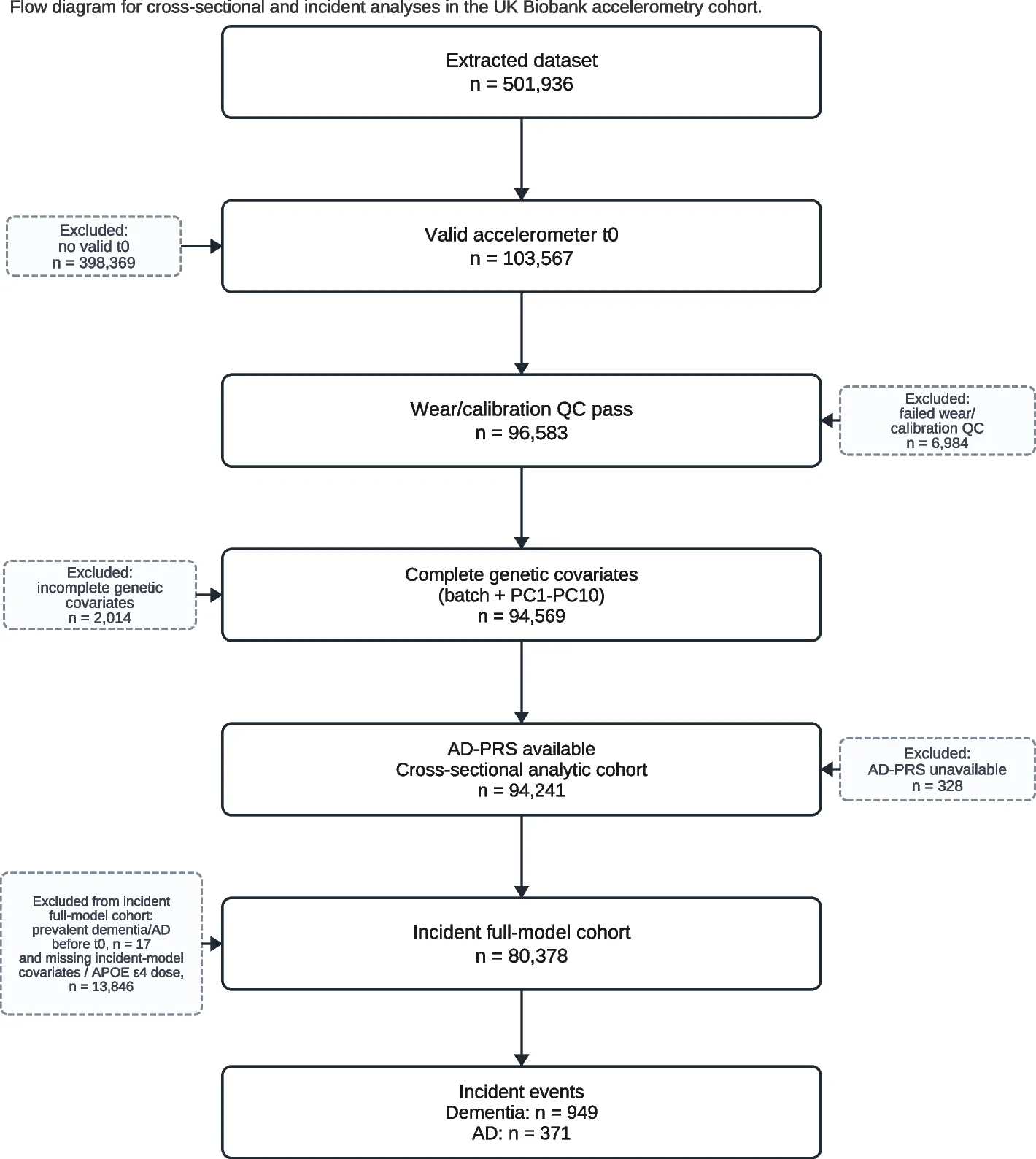
Participant flow and exclusion summary. Flow diagram of inclusion, exclusion, and quality-control steps for the cross-sectional analytic cohort and the incident full-model cohort, including event counts

We began with 501,936 participants in the extracted dataset. Inclusion required a valid accelerometer end date (t0), passing wear/calibration quality control, complete genetic covariates (genotyping batch and principal components PC1-PC10), and availability of the AD-PRS variable used in the current dataset release. Participants with prevalent dementia/AD prior to t0 were excluded. The final cross-sectional analytic cohort comprised 94,241 participants (Fig. 1). Sample size was determined by data availability; we included all eligible participants meeting the prespecified QC and eligibility criteria (no a priori power calculation).

For incident analyses, participants were required to be free of dementia and AD prior to t0 and were followed until the earliest of incident outcome, death, loss to follow-up, or administrative censoring. Because UK Biobank accelerometry was collected in a selected subcohort, we prespecified sensitivity analyses to evaluate robustness after excluding early events, to assess competing mortality, and to examine selection into the accelerometry sample; the latter was addressed using inverse probability weighting (IPW).

### Accelerometry-derived circadian phenotypes

Raw tri-axial wrist accelerometry in UK Biobank was collected at 100 Hz for 7 consecutive days and processed using established procedures, including autocalibration using local gravity, removal of gravity and sensor noise, and identification of non-wear episodes, yielding calibrated acceleration summaries and epoch-level time series suitable for circadian and sleep research [4–6]. We used the derived 24-h mean acceleration profile and the 5-s epoch time series (Field 90,004) to characterize nonparametric circadian rest–activity rhythms, focusing on amplitude (RA), phase timing (L5 and M10 start hour), and rhythm stability/fragmentation (IS and IV). These metrics index the timing and consolidation of the 24-h activity pattern rather than direct research-grade measures of sleep staging, wake after sleep onset, or polysomnographic sleep duration.

UK Biobank releases both raw accelerometry signals and standardized derived variables generated using a common processing pipeline. In our primary analyses, we treated relative amplitude (RA) and the timing metrics (L5/M10 and their start hours) as UK Biobank–derived circadian rest–activity rhythm (CRAR) phenotypes to maximize comparability with prior UK Biobank work [4–6]. By contrast, to make explicit the regular time-series construction and quality thresholds used for stability/fragmentation metrics, we computed interdaily stability (IS) and intradaily variability (IV) from the 5-s epoch time series (Field 90,004) after binning, as described below [4–6]. This separation clarifies which CRAR metrics are platform-derived versus study-derived and improves reproducibility.

From the 24-h mean acceleration profile computed from 7-day wrist accelerometry, we derived:

- L5 start hour: start time of the least active 5-h window (0–23).
- M10 start hour: start time of the most active 10-h window (0–23).
- Relative amplitude (RA): (M10—L5)/(M10 + L5).

L5 and M10 were computed as rolling windows across the 24-h day and summarized as the integer start hour (0–23) of the least-active 5-h period and the most-active 10-h period, respectively. L5 timing approximates when the least-active part of the day occurs, often overlapping with habitual nocturnal rest, but it is not a direct measure of sleep onset or sleep duration. A later L5 start therefore indicates that the least-active window occurs later in the 24-h cycle, consistent with delayed or redistributed inactivity. M10 timing reflects the phase of peak daytime activity rather than wake time itself. RA was calculated from the mean activity levels during these windows; lower RA indicates a weaker contrast between daytime activity and nighttime inactivity and may arise from reduced daytime activity, greater nighttime activity, daytime inactivity/napping, or more fragmented rest–activity organization.

To quantify stability and fragmentation, we derived:

- Interdaily stability (IS): capturing day-to-day regularity of the 24-h pattern.
- Intradaily variability (IV): capturing within-day fragmentation.

IS and IV were derived from raw 5-s epoch time-series (UK Biobank Field 90,004), aggregated into 30-min bins to form a regular time series [4–6]. We required at least 240 bins (at least 5 days) to ensure adequate coverage.

We selected 30-min binning to obtain a regular time series and to reduce sensitivity to short periods of non-wear or transient spikes. The ≥ 240-bin threshold (≥ 5 days) was chosen to balance sample retention with estimate stability for IS/IV; the corresponding quality control flow is shown in Fig. 1 and Supplementary Figs. S3–S4.

### Genetic exposures

AD-PRS: standardized (mean 0, SD 1) within the analytic sample, corresponding to the dataset-provided UK Biobank standard AD PRS variable (field 26,206) used in the current release. We did not perform de novo SNP-level PRS construction within this project; accordingly, SNP inclusion/exclusion and weighting were determined by the upstream UK Biobank PRS resource rather than by the present analysis pipeline.

*APOE* ε4 dose: 0/1/2 ε4 alleles based on rs429358 and rs7412 genotype combinations [7].

To mitigate population structure confounding, all genetic models included genotyping batch and the first ten genetic principal components (PC1–PC10), and we conducted a European-only restriction sensitivity analysis (ancestry_group = 1) to evaluate robustness to ancestry heterogeneity (Table S3). We used AD-PRS and *APOE* ε4 dose as complementary, not interchangeable, genetic exposures: *APOE* captures a major-effect locus, whereas AD-PRS captures broader polygenic susceptibility beyond *APOE*.

### Covariates and missing data

Basic model covariates were age at t0, sex, Townsend deprivation index, genotyping batch, and PC1-PC10. Fully adjusted models further included BMI, smoking status, alcohol intake frequency, bipolar/major depression status, shift work, and night shift work. These covariates were taken from available UK Biobank baseline assessment or linked derived variables carried into the analysis-ready table.

Fully adjusted model: basic covariates plus BMI, smoking, alcohol intake frequency, depression, shift work, and night shift work.

To minimize sample attrition, additional covariates were handled via mean/mode imputation plus missingness indicators (single imputation with indicator approach). This was chosen to preserve sample size and maintain a consistent analytic population across the multiple sensitivity analyses, but we acknowledge in the Limitations section that this pragmatic approach may introduce bias if missingness is informative.

The imputation-plus-indicator approach was chosen because it preserves sample size and maintains a consistent analytic population across models while allowing missingness patterns to be explicitly represented. Continuous covariates were imputed to the cohort mean and categorical variables to the modal category, with a corresponding missingness indicator for each imputed variable included in fully adjusted models.

### Incident dementia and incident AD

Incident outcomes were defined from UK Biobank linked health-record first-occurrence ICD-10 dates after t0; these reflect clinically coded diagnoses from routine data linkage rather than research-adjudicated dementia phenotypes:

- Dementia: F00/F01/F02/F03/G30/G31 (earliest date).
- AD (broad): G30 and/or F00 (earliest date).
- AD (strict, sensitivity): G30 only (earliest date).

Follow-up was censored at an administrative end date of 2025–07-01 in primary analyses to match the available extracted dataset. Time-to-event was calculated as (event/censor date—t0)/365.25. In competing-risk sensitivity analyses, deaths after t0 were additionally treated as competing events.

We used UK Biobank first-occurrence fields to identify incident events after t0, and we summarized the ICD-10 code lists used for broad and strict endpoint definitions in Table S6. Participants with an event date prior to t0 for the corresponding outcome were treated as prevalent cases and excluded from incident analyses.

### Statistical analysis

Cross-sectional outcomes were analyzed by linear regression; results were reported as standardized beta (SD units) with 95% confidence intervals. Incident outcomes were analyzed using Cox proportional hazards models with Breslow ties [14] and the fully adjusted covariate set. Continuous circadian predictors were modeled per SD.

For cross-sectional models, outcomes were standardized and models were fit separately for AD-PRS and *APOE* ε4 dose. We reported two covariate sets (basic and fully adjusted) to evaluate confounding sensitivity. For incident models, we modeled genetic predictors (AD-PRS per SD, *APOE* ε4 dose per allele) and circadian predictors (per SD) in fully adjusted models, and we summarized key-term estimates in Table 5 and Figs. 3 and 4.

Sensitivity analyses were prespecified to evaluate robustness after excluding early incident events, to assess competing risk, to examine potential confounding by overall activity, and to address selection into the accelerometry subcohort. We repeated incident models after excluding events within 1, 2, and 5 years after t0 (lag analyses). We evaluated death as a competing event using both cause-specific Cox and Fine–Gray subdistribution hazards models. To assess whether RA primarily reflected general activity/health status, we additionally adjusted for overall activity level (mean acceleration). Selection bias was examined using inverse probability weighting (IPW) based on the probability of inclusion in the accelerometry cohort. Finally, we tested endpoint robustness using a stricter AD definition (G30 only) in addition to the broad definition (G30 or F00).

To test whether the RA association differed across genetic-risk levels, we added multiplicative interaction terms (RA × AD-PRS and RA × *APOE* ε4 dose) in fully adjusted incident Cox models for incident dementia and broad AD. We also conducted exploratory cross-sectional sensitivity analyses using *APOE* genotype categories (ε2/4, ε3/4, and ε4/4 vs non-carriers) and repeated those models after excluding ε2/4 participants.

### Multiple testing (IS/IV)

IS/IV analyses involved 8 primary tests (2 outcomes × 2 exposures × 2 models). We report Bonferroni (alpha = 0.00625) and Benjamini–Hochberg false discovery rate (BH-FDR) q-values [15]. Unless explicitly stated otherwise, *p*-values in the remaining cross-sectional, incident, and sensitivity analyses are nominal and are interpreted as part of a robustness framework rather than as a family-wise multiplicity-controlled testing scheme.

## Results

### Cohort assembly

From 501,936 participants in the extracted dataset, 103,567 had valid accelerometry t0 and 96,583 passed wear/calibration quality control. After requiring complete genetic covariates (*n* = 94,569) and AD-PRS availability (*n* = 94,241), the final cross-sectional analytic cohort comprised 94,241 participants (Fig. 1). For prospective full-model analyses, 80,378 participants remained after excluding prevalent dementia/AD before t0 and participants without complete incident-model covariates. Compared with UK Biobank participants not included in the accelerometry analytic cohort, included participants were slightly younger and leaner and had lower proportions of current smoking and less frequent heavy alcohol intake, consistent with healthier selection into accelerometry participation (Table S9). Baseline characteristics across AD-PRS quartiles are summarized in Table 1.

**Table 1:** Baseline characteristics by AD-PRS quartile

Characteristic	Category	Q1	Q2	Q3	Q4
Participants, n		23,559	23,559	23,571	23,552
Age at accelerometry end (years)		62.460 (7.855)	62.433 (7.877)	62.371 (7.878)	62.229 (7.762)
Townsend deprivation index		−1.713 (2.838)	−1.716 (2.831)	−1.715 (2.809)	−1.781 (2.793)
Body mass index, kg/m^2^		26.791 (4.570)	26.729 (4.544)	26.685 (4.466)	26.607 (4.502)
Mean acceleration, mg		27.911 (8.333)	27.960 (8.185)	27.992 (8.381)	28.294 (8.508)
Relative amplitude		0.856 (0.070)	0.856 (0.071)	0.856 (0.072)	0.858 (0.067)
L5 mean activity, mg		3.359 (1.648)	3.371 (1.698)	3.373 (1.676)	3.369 (1.694)
M10 mean activity, mg		46.773 (15.172)	46.853 (14.908)	46.863 (15.354)	47.361 (15.352)
L5 start hour, h		2.823 (5.953)	2.960 (6.144)	2.829 (5.988)	2.866 (6.063)
M10 start hour, h		8.596 (1.551)	8.591 (1.582)	8.597 (1.550)	8.580 (1.546)
Wear duration, days		6.660 (0.657)	6.661 (0.654)	6.660 (0.653)	6.658 (0.656)
Non-wear duration, days		0.298 (0.587)	0.298 (0.586)	0.301 (0.591)	0.301 (0.592)
Sex	Female	13,334 (56.6%)	13,122 (55.7%)	13,214 (56.1%)	13,251 (56.3%)
	Male	10,225 (43.4%)	10,437 (44.3%)	10,357 (43.9%)	10,301 (43.7%)
Smoking status	Never	13,330 (56.6%)	13,386 (56.8%)	13,373 (56.7%)	13,585 (57.7%)
	Previous	8575 (36.4%)	8423 (35.8%)	8469 (35.9%)	8355 (35.5%)
	Current	1582 (6.7%)	1691 (7.2%)	1680 (7.1%)	1552 (6.6%)
	Prefer not to answer	61 (0.3%)	53 (0.2%)	41 (0.2%)	53 (0.2%)
Alcohol intake frequency	Never	1325 (5.6%)	1328 (5.6%)	1394 (5.9%)	1290 (5.5%)
	Prefer not to answer	13 (0.1%)	8 (0.0%)	6 (0.0%)	9 (0.0%)
	Daily or almost daily	5374 (22.8%)	5446 (23.1%)	5461 (23.2%)	5290 (22.5%)
	3–4 times/week	6136 (26.0%)	6102 (25.9%)	6091 (25.8%)	6201 (26.3%)
	1–2 times/week	5862 (24.9%)	5823 (24.7%)	5953 (25.3%)	5960 (25.3%)
	1–3 times/month	2566 (10.9%)	2612 (11.1%)	2449 (10.4%)	2606 (11.1%)
	Special occasions only	2271 (9.6%)	2234 (9.5%)	2209 (9.4%)	2189 (9.3%)
Bipolar/major depression status	Bipolar II disorder	36 (0.2%)	40 (0.2%)	26 (0.1%)	32 (0.1%)
	Probable recurrent major depression, severe	482 (2.0%)	464 (2.0%)	432 (1.8%)	451 (1.9%)
	Probable recurrent major depression, moderate	862 (3.7%)	811 (3.4%)	803 (3.4%)	829 (3.5%)
	Single probable major depression episode	438 (1.9%)	436 (1.9%)	521 (2.2%)	450 (1.9%)
	No bipolar or depression	4724 (20.1%)	4663 (19.8%)	4664 (19.8%)	4658 (19.8%)
	Bipolar I disorder	37 (0.2%)	26 (0.1%)	29 (0.1%)	42 (0.2%)
Shift work	Never/rarely	12,443 (52.8%)	12,542 (53.2%)	12,563 (53.3%)	12,846 (54.5%)
	Sometimes	870 (3.7%)	860 (3.7%)	892 (3.8%)	878 (3.7%)
	Usually	237 (1.0%)	220 (0.9%)	217 (0.9%)	229 (1.0%)
	Do not know	14 (0.1%)	16 (0.1%)	15 (0.1%)	16 (0.1%)
	Always	811 (3.4%)	767 (3.3%)	824 (3.5%)	781 (3.3%)
	Prefer not to answer	7 (0.0%)	4 (0.0%)	3 (0.0%)	10 (0.0%)
Night shift work	Prefer not to answer	5 (0.0%)	3 (0.0%)	1 (0.0%)	9 (0.0%)
	Sometimes	533 (2.3%)	530 (2.2%)	553 (2.3%)	542 (2.3%)
	Usually	141 (0.6%)	143 (0.6%)	155 (0.7%)	145 (0.6%)
	Always	242 (1.0%)	207 (0.9%)	241 (1.0%)	238 (1.0%)
	Do not know	4 (0.0%)	2 (0.0%)	4 (0.0%)	3 (0.0%)
	Never/rarely	1014 (4.3%)	982 (4.2%)	997 (4.2%)	977 (4.1%)
APOE ε4 dose	0 ε4 alleles	19,510 (82.8%)	18,788 (79.7%)	15,618 (66.3%)	3938 (16.7%)
	1 ε4 allele	598 (2.5%)	1333 (5.7%)	4393 (18.6%)	14,525 (61.7%)
	2 ε4 alleles	2 (0.0%)	14 (0.1%)	58 (0.2%)	1709 (7.3%)

Values are mean (SD) for continuous variables and n (%) for categorical variables. Quartiles are ordered from lowest (Q1) to highest (Q4) AD-PRS. RA indicates the contrast between daytime activity and nighttime inactivity

### Cross-sectional associations: RA and phase timing

**Fig. 2:**
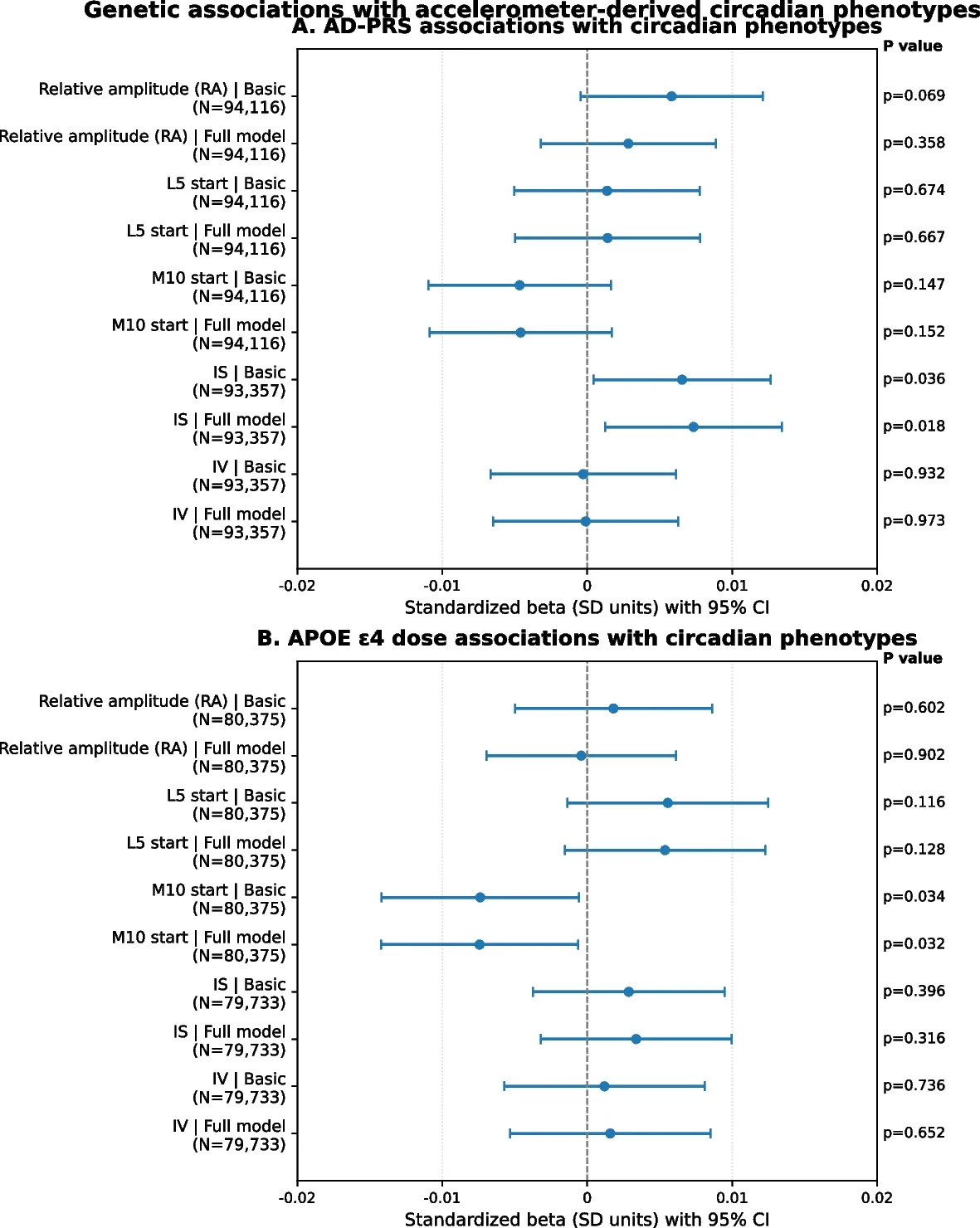
Genetic associations with circadian phenotypes. Standardized beta (95% CI) from linear regression for AD-PRS and *APOE* ε4 dose across RA, L5/M10 start hour, IS, and IV in basic and fully adjusted models. Earlier M10 indicates earlier peak daytime activity timing. IS/IV *p*-values are nominal; multiplicity results are reported in Table 4 and Table S1

In PRS models, AD-PRS showed no robust association with RA in fully adjusted models (standardized beta = 0.0028; *p* = 0.358), and associations with L5/M10 start hour were small and not statistically robust across covariate sets (Table 2; Fig. 2; Fig. S1). In APOE models, APOE ε4 dose was associated with earlier M10 timing (standardized beta = −0.0074; *p* = 0.032), while associations with RA and L5 timing were not significant (Table 3; Fig. 2; Fig. S2). Earlier M10 reflects an earlier peak daytime activity window rather than wake time itself.

**Table 2:** AD-PRS associations with circadian amplitude and timing

Outcome	Model	n	Exposure	Standardized β (95% CI)	*P* value
Relative amplitude (RA)	Basic	94116	AD-PRS	0.006 (−0.000461 to 0.012110)	0.069
Relative amplitude (RA)	Fully adjusted	94116	AD-PRS	0.003 (−0.003205 to 0.008875)	0.358
L5 start hour	Basic	94116	AD-PRS	0.001 (−0.005026 to 0.007769)	0.674
L5 start hour	Fully adjusted	94116	AD-PRS	0.001 (−0.004986 to 0.007796)	0.667
M10 start hour	Basic	94116	AD-PRS	−0.005 (−0.010959 to 0.001635)	0.147
M10 start hour	Fully adjusted	94116	AD-PRS	−0.005 (−0.010873 to 0.001694)	0.152

Linear regression results are shown as standardized β (95% CI). Basic models adjust for age at t0, sex, Townsend deprivation index, genotyping batch, and PC1–PC10. Fully adjusted models additionally include BMI, smoking, alcohol intake frequency, bipolar/major depression status, shift work, and night shift work

**Table 3:** APOE ε4 dose associations with circadian amplitude and timing

Outcome	Model	n	Exposure	Standardized β (95% CI)	*P* value
Relative amplitude (RA)	Basic	80375	APOE ε4 dose	0.002 (−0.004989 to 0.008611)	0.602
Relative amplitude (RA)	Fully adjusted	80375	APOE ε4 dose	−0.000 (−0.006952 to 0.006130)	0.902
L5 start hour	Basic	80375	APOE ε4 dose	0.006 (−0.001364 to 0.012483)	0.116
L5 start hour	Fully adjusted	80375	APOE ε4 dose	0.005 (−0.001550 to 0.012282)	0.128
M10 start hour	Basic	80375	APOE ε4 dose	−0.007 (−0.014197 to −0.000572)	0.034
M10 start hour	Fully adjusted	80375	APOE ε4 dose	−0.007 (−0.014227 to −0.000633)	0.032

Linear regression results are shown as standardized β (95% CI). Earlier M10 indicates an earlier peak daytime activity window rather than wake-up time itself

In exploratory genotype-category sensitivity analyses, ε3/4 heterozygotes showed no stable differences in RA or phase timing compared with non-carriers, whereas ε4/4 homozygotes showed a modest later L5 start time (approximately 18.3 min; –*p* = 0.036). These exploratory category-based findings do not support a simple interpretation that the primary per-allele APOE result reflects shorter nocturnal sleep duration; rather, they suggest a small shift in rest–activity phase, with no stable signal among heterozygotes. The pattern was similar after excluding ε2/4 participants, indicating that the primary per-allele APOE result was not driven by ε2/4 genotypes (Table S2).

### IS/IV associations (full dataset)

In full-dataset IS/IV analyses (AD-PRS *n* = 93,357; *APOE n* = 79,733), AD-PRS showed a very small nominal association with IS (standardized beta = 0.00733; nominal *p* = 0.018). However, across the 8 IS/IV tests, no association remained significant after Bonferroni correction (alpha = 0.00625) or BH-FDR (minimum q≈0.144) (Table 4; Supplementary Table S1). Overall, effect sizes for IS/IV were extremely small, and patterns were consistent with limited cross-sectional signal for AD polygenic risk in these fragmentation/stability metrics.

**Table 4:** IS/IV associations

Outcome	Model	n	Exposure	Standardized β (95% CI)	*P* value (nominal)
Interdaily stability (IS)	Basic	93,357	AD-PRS	0.007 (0.000430 to 0.012665)	0.036
Interdaily stability (IS)	Fully adjusted	93,357	AD-PRS	0.007 (0.001233 to 0.013428)	0.018
Interdaily stability (IS)	Basic	79,733	APOE ε4 dose	0.003 (−0.003747 to 0.009474)	0.396
Interdaily stability (IS)	Fully adjusted	79,733	APOE ε4 dose	0.003 (−0.003215 to 0.009962)	0.316
Intradaily variability (IV)	Basic	93,357	AD-PRS	−0.000 (−0.006673 to 0.006119)	0.932
Intradaily variability (IV)	Fully adjusted	93,357	AD-PRS	−0.000 (−0.006491 to 0.006271)	0.973
Intradaily variability (IV)	Basic	79,733	APOE ε4 dose	0.001 (−0.005732 to 0.008110)	0.736
Intradaily variability (IV)	Fully adjusted	79,733	APOE ε4 dose	0.002 (−0.005316 to 0.008496)	0.652

Linear regression results are shown as standardized β (95% CI). IS and IV analyses used a QC threshold of at least 240 30-min bins (approximately 5 days). *P* values in this table are nominal; multiplicity-adjusted results are provided in Table S1

### Incident dementia and incident AD

After excluding prevalent dementia/AD prior to t0, 80,378 participants were included for incident analyses through the administrative end date of 2025–07-01. There were 949 incident dementia events and 371 incident AD events during follow-up anchored to the accelerometry end date (t0).

**Fig. 3:**
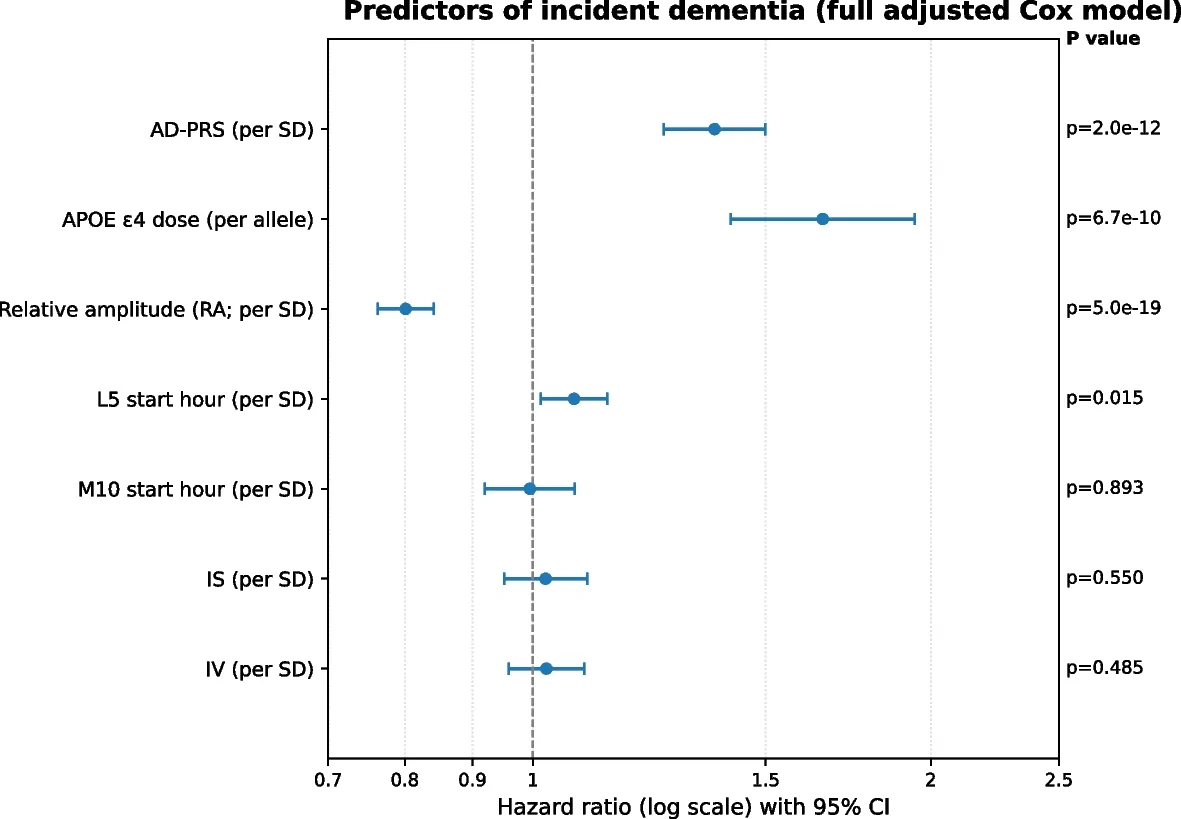
Predictors of incident dementia. Hazard ratios (log scale) from fully adjusted Cox models for AD-PRS, *APOE* ε4 dose, and circadian metrics (per SD). Full models adjust for age at t0, sex, Townsend deprivation index, genotyping batch, PC1–PC10, BMI, smoking, alcohol intake frequency, depression, shift work, and night shift work

**Fig. 4:**
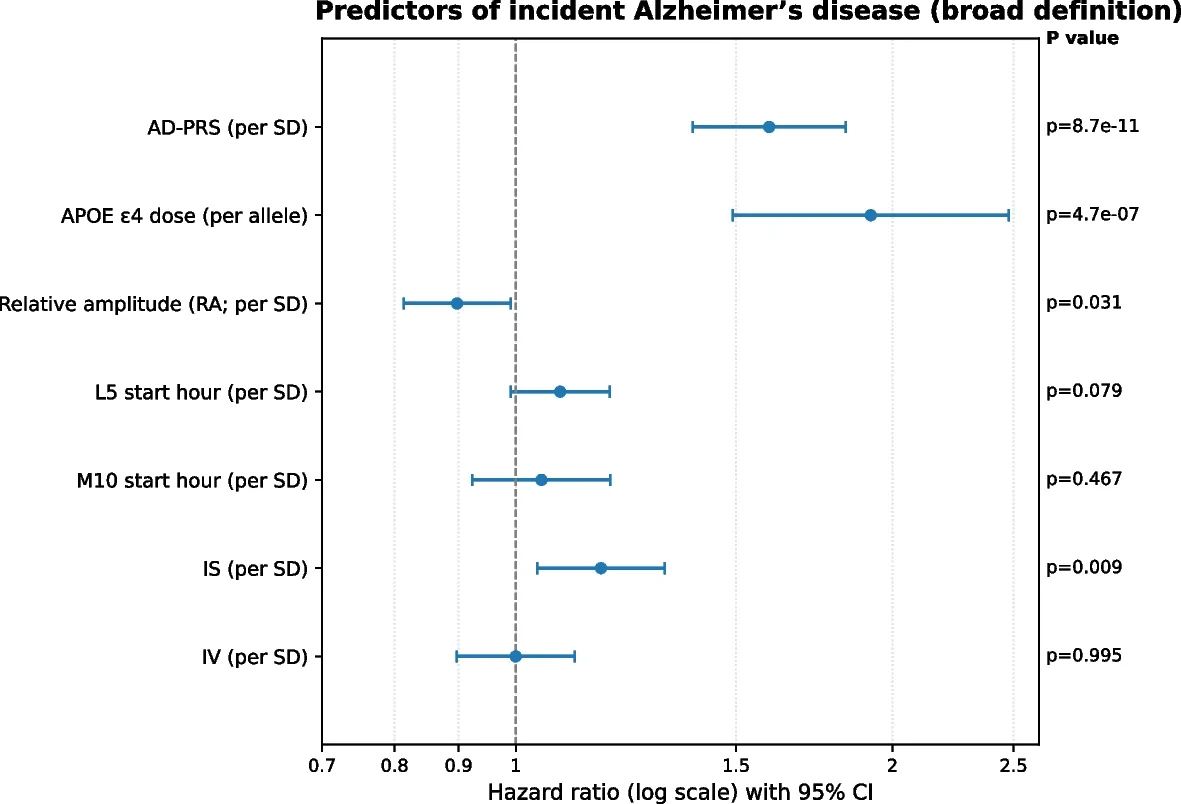
Predictors of incident Alzheimer’s disease. Hazard ratios (log scale) from fully adjusted Cox models for AD-PRS, *APOE* ε4 dose, and circadian metrics (per SD). Full models adjust for age at t0, sex, Townsend deprivation index, genotyping batch, PC1–PC10, BMI, smoking, alcohol intake frequency, depression, shift work, and night shift work

In genetics-only Cox models, AD-PRS and APOE ε4 dose strongly predicted both incident dementia and incident AD. In fully adjusted models including circadian predictors, each 1-SD higher RA was associated with lower risk of incident dementia (HR = 0.801 per SD; *p* = 4.99 × 10^-19^) and incident AD (HR = 0.898; *p* = 0.031); equivalently, lower RA corresponded to higher risk. L5 start hour showed a modest positive association with incident dementia, whereas other circadian metrics showed smaller or less consistent associations. IS was not associated with incident dementia in the full-adjusted model (HR = 1.022; *p* = 0.550) but showed a modest association with incident AD (HR = 1.170; *p* = 0.0088). Key-term Cox estimates are summarized in Table 5 and Figs. 3 and 4; full model outputs are provided in Supplementary Table S5.

**Table 5:** Key terms from incident models

Outcome	Model block	Term	HR (95% CI)	*P* value
Incident dementia	Genetics	AD-PRS (per SD)	1.372 (1.256, 1.499)	< 0.001
Incident dementia	Genetics	APOE ε4 dose (per allele)	1.657 (1.411, 1.945)	< 0.001
Incident dementia	RA	RA (per SD)	0.801 (0.763, 0.841)	< 0.001
Incident dementia	L5_start_hour	L5_start_hour (per SD)	1.074 (1.014, 1.138)	0.015
Incident dementia	M10_start_hour	M10_start_hour (per SD)	0.995 (0.920, 1.076)	0.893
Incident dementia	IS	IS (per SD)	1.022 (0.951, 1.099)	0.550
Incident dementia	IV	IV (per SD)	1.024 (0.958, 1.094)	0.485
Incident AD	Genetics	AD-PRS (per SD)	1.594 (1.385, 1.836)	< 0.001
Incident AD	Genetics	APOE ε4 dose (per allele)	1.922 (1.491, 2.478)	< 0.001
Incident AD	RA	RA (per SD)	0.898 (0.814, 0.990)	0.031
Incident AD	L5_start_hour	L5_start_hour (per SD)	1.085 (0.991, 1.188)	0.079
Incident AD	M10_start_hour	M10_start_hour (per SD)	1.048 (0.923, 1.190)	0.467
Incident AD	IS	IS (per SD)	1.170 (1.040, 1.315)	0.009
Incident AD	IV	IV (per SD)	1.000 (0.897, 1.114)	0.995

Fully adjusted Cox model estimates for genetic exposures and circadian metrics. Hazard ratios are reported per 1-SD higher value for continuous predictors. For RA, an HR below 1.0 means that lower RA corresponds to higher risk

In incident interaction analyses, we found no evidence that the association of RA with incident dementia or broad AD differed by AD-PRS or APOE ε4 dose (all interaction *p* values ≥ 0.362; Table S7).

### Sensitivity analyses

Lagged analyses excluding events within 1, 2, and 5 years after t0 yielded directionally consistent associations for genetic predictors and RA, supporting robustness after excluding near-term post-t0 diagnoses (Supplementary Figures S8–S10). These lagged analyses reduce the influence of early diagnostic or prodromal events but cannot exclude long preclinical neurodegenerative processes.

In competing-risk analyses treating death as a competing event, higher RA remained associated with lower risk of incident dementia and incident AD in both cause-specific Cox and Fine–Gray models, with consistent post-t0 event-count accounting (cause-specific vs Fine–Gray HRs: dementia 0.786 vs 0.801; broad AD 0.870 vs 0.898) (Figure S12).

After additional adjustment for overall activity level, the association between RA and incident dementia remained statistically robust (HR = 0.827; *p* = 3.39 × 10^-11^), whereas the association with incident AD attenuated (HR = 0.926; *p* = 0.185) (Figure S13).

Inverse probability weighted Cox models to account for selection into the accelerometry cohort produced similar estimates for RA (incident dementia *p* = 2.03 × 10^–17^; incident AD *p* = 0.023), indicating that the primary RA findings were not materially altered by this selection-weighting sensitivity analysis (Figure S14).

Endpoint sensitivity analyses using a strict AD definition (G30 only) showed patterns consistent with the broad definition (G30 or F00) (Figure S11).

## Discussion

In this UK Biobank analysis integrating AD-PRS and APOE ε4 dose with accelerometry-derived nonparametric circadian rest–activity rhythm (CRAR) phenotypes, we observed a consistent pattern across cross-sectional and prospective models anchored at the accelerometry end date (t0). Cross-sectionally, AD-PRS showed essentially null association with RA in fully adjusted models, while APOE ε4 dose was associated with a modestly earlier M10 start hour. Prospectively, lower RA corresponded to higher risk of incident dementia and showed a weaker association with incident AD. The RA–dementia signal remained stable across lagged exclusions, competing-risk models, and selection weighting, and it remained significant after additional adjustment for overall activity. By contrast, the RA–incident AD association attenuated after overall-activity adjustment. We additionally found no evidence that the RA association differed by AD-PRS or APOE ε4 dose in formal incident interaction analyses.

A central consideration in UK Biobank dementia research is outcome ascertainment. First-occurrence ICD-10 definitions enable scale but may misclassify etiologic subtypes; validation work supports stronger performance for all-cause dementia than for AD subtype coding [16]. Accordingly, we interpret dementia as the primary endpoint and regard AD subtype analyses as supportive, emphasizing patterns that persist across broad versus strict definitions and across sensitivity analyses.

Our findings align with accumulating wearable evidence that weaker rest–activity rhythmicity predicts adverse cognitive and dementia outcomes. In UK Biobank, lower RA and related CRAR disruptions have been linked to incident dementia as well as to cognitive and neuroimaging correlates [17], and impaired 24-h activity patterns have been associated with increased risk of subsequent AD and other neurodegenerative outcomes [18]. Beyond UK Biobank, circadian rest–activity rhythm disruption has predicted delirium and subsequent dementia progression in clinical cohorts [19], and a 1-SD decrement in RA was associated with higher incident dementia risk in the ARIC study [20]. Prior work has also shown that demographic and genetic factors can modify associations of CRAR metrics with cognition [21], which underscores the value of our formal interaction testing between RA and genetic risk in the present analysis.

RA reflects the contrast between daytime activity and nighttime inactivity and is therefore expected to correlate with activity volume and general health status. Lower RA can arise from reduced daytime activity, greater nighttime activity, daytime inactivity/napping, or more fragmented day–night organization. When we additionally adjusted for overall activity, the RA–dementia association remained strongly significant, suggesting that rhythm consolidation carries information beyond activity volume for all-cause dementia in this cohort. In contrast, the RA–incident AD association became less robust, which may reflect both greater etiologic misclassification for AD subtyping in routinely coded data [16] and overlap with broader activity-related health. We therefore interpret RA primarily as a robust behavioral risk marker for subsequent dementia rather than as a disease-specific mechanistic biomarker for AD.

Circadian disruption can be both an early manifestation of neurodegeneration and a plausible contributor to disease biology through sleep-dependent proteostasis, metabolic regulation, and immune signaling [1, 2, 22–26]. In our cross-sectional models, the absence of a meaningful association between AD-PRS and RA argues against a simple pathway in which greater polygenic susceptibility strongly shapes week-long behavioral rhythm amplitude in mid-to-late adulthood. Instead, RA likely acts as an integrative digital marker reflecting a mixture of modifiable behaviors (sleep regularity, light–dark structure, daily routines), comorbidity-related constraints on activity, and potentially early neurobiological change.

The APOE-related timing result also warrants cautious interpretation. In the per-allele model, APOE ε4 dose was associated with slightly earlier M10 timing, which reflects an earlier peak daytime activity window rather than wake time itself. However, genotype-category analyses did not show a stable graded pattern across ε3/4 and ε4/4 groups. Heterozygotes did not show consistent differences, whereas homozygotes showed a modest later L5 start time rather than a cleanly earlier M10 shift. These exploratory results therefore do not support a simple conclusion that APOE ε4 homozygotes have shorter nocturnal sleep; instead, they suggest small and potentially heterogeneous changes in rest–activity phase during the preclinical or prodromal period.

The divergence between *APOE* and AD-PRS is informative. *APOE* ε4 is a major-effect locus with pleiotropic biology, whereas AD-PRS summarizes distributed genome-wide susceptibility [7–10, 27]. Notably, both AD-PRS and *APOE* ε4 dose strongly predicted incident dementia and incident AD, indicating that genetic susceptibility was expressed primarily through disease risk rather than through large baseline differences in week-long circadian behavior.

Taken together, our results support a cautious framework in which stable genetic susceptibility and wearable-derived behavioral rhythms capture different aspects of dementia risk. The formal interaction analyses did not support a stronger protective association of high RA in genetically higher-risk groups. Future work should therefore prioritize repeated accelerometry, richer contextual data (for example light exposure, chronotype, medication, and sleep disorders), and biomarker linkage to distinguish modifiable behavioral signatures from prodromal neurodegenerative change.

### Strengths

Key strengths include population-scale 7-day accelerometry with standardized processing and rigorous quality control, integrated with both major-effect (APOE) and polygenic AD risk and long-term linkage to electronic health records [4–6, 11, 12]. We anchored time zero at the accelerometry end date to align exposure assessment with prospective risk and reduce time-origin ambiguities. By distinguishing platform-derived CRAR metrics (RA and phase timing) from study-derived stability/fragmentation measures (IS/IV computed from the epoch time series with explicit binning and quality thresholds), we aimed to maximize comparability while making the most assumption-sensitive steps transparent.

We also implemented a prespecified, bias-oriented sensitivity battery that mirrors major validity concerns in wearable epidemiology. Lagged exclusions assessed sensitivity to near-term post-t0 diagnoses; competing-risk models accounted for the high background mortality burden; inverse probability weighting addressed selection into the accelerometry subcohort; and activity-adjusted models tested whether RA carries information beyond activity volume [28–32]. The convergence of results across these stress tests strengthens confidence that the RA–dementia association is not a single-model artifact.

### Implications and future work

From a translational perspective, combining scalable wearables with genetic profiling may support a pragmatic approach to risk stratification: genetics captures relatively stable susceptibility, whereas CRAR metrics capture current behavioral and physiological organization that may be modifiable. Such an approach could help enrich prevention studies or prioritize biomarker assessment when PET or CSF testing are not feasible at scale. At the same time, PRS transferability across ancestries and the social patterning of wearable phenotypes warrant careful evaluation to avoid widening health disparities [9, 10].

Future work should (i) replicate findings across independent cohorts using harmonized actigraphy pipelines; (ii) examine trajectories of CRAR metrics and their coupling with cognition and neuroimaging over time; (iii) integrate timing of activity and sleep regularity with light exposure and chronotype to move from risk markers toward actionable targets; and (iv) test whether interventions that strengthen circadian organization can shift intermediate biomarkers or cognitive trajectories. Potential effect modification by sex, age, ancestry, and *APOE* genotype also remains an important target for future studies [21, 33–35].

### Limitations

Several limitations merit emphasis. First, this study is observational; residual confounding and long preclinical neurodegenerative processes cannot be fully excluded. Although lagged analyses reduce the influence of near-term diagnoses, they do not rule out longer prodromal processes that may already have affected baseline rest–activity patterns. Wearable-derived rhythm metrics are correlated with mobility, comorbidity burden, medication use, and socioeconomic factors, and some influences may not be completely captured by available covariates. We therefore interpret RA primarily as a behavioral risk marker rather than a proven causal driver of dementia.

Second, accelerometry was assessed over a single 7-day window, which may not fully represent long-term habitual circadian behavior; estimates can vary with season, weekday structure, travel, and transient illness. Although UK Biobank accelerometry processing is standardized, measurement error and imperfect separation of circadian amplitude from overall activity volume remain important considerations [4–6, 36, 37].

Third, incident dementia and AD were defined using first-occurrence ICD-10 dates from linked routine health records, which may be delayed or incomplete and differ in accuracy across dementia subtypes. Prior validation work indicates stronger performance for all-cause dementia than for AD subtype ascertainment, consistent with our more cautious interpretation of AD endpoint results [16]. Fourth, the number of incident events was small relative to the analytic cohort size, particularly for incident AD (371 events among 80,378 participants), which limited statistical precision for AD-specific, genotype-category, and interaction analyses. Fifth, we used single imputation with missingness indicators for covariates to preserve sample size and maintain a consistent analytic population across models. Although pragmatic, this approach can introduce bias if missingness is informative; accordingly, the adjusted estimates should be interpreted with appropriate caution. Finally, UK Biobank participants are generally healthier than the source population and accelerometry participation represents an additional selection step; we attempted to mitigate this via inverse probability weighting, but generalizability to other settings and ancestry groups should be assessed via external replication.

## Conclusions

In this UK Biobank cohort, AD polygenic risk showed minimal cross-sectional association with accelerometry-derived circadian amplitude or phase, whereas APOE ε4 dose was associated with slightly earlier peak daytime activity timing. Prospectively, lower circadian amplitude (RA) corresponded to higher dementia risk across multiple bias-oriented sensitivity analyses.

We found no evidence that the RA association differed by AD-PRS or APOE ε4 dose, and the RA–incident AD association weakened after additional control for overall activity. These findings support RA as a behavioral marker of subsequent dementia risk while underscoring the need for cautious interpretation of disease specificity and for further validation with repeated wearable assessment and biomarker-linked studies.

## Supplementary Information

Additional file 1: Supplementary Figures S1–S14. Description: Additional figures supporting sensitivity analyses and ancillary results referenced in the main text. Fig. S1. AD-PRS associations with circadian phenotypes. Standardized beta (95% CI) for RA, L5/M10 start hour, IS, and IV in basic and fully adjusted models. Fig. S2. *APOE* ε4 dose associations with circadian phenotypes. Standardized beta (95% CI) for RA, L5/M10 start hour, IS, and IV in basic and fully adjusted models. Fig. S3. European-only sensitivity analysis. AD-PRS and *APOE* ε4 dose associations with RA and phase timing restricted to ancestry_group = 1. Fig. S4A. IS/IV associations for AD-PRS. Standardized beta (95% CI) in basic and fully adjusted models; QC required ≥ 240 30-min bins. Fig. S4B. IS/IV associations for *APOE* ε4 dose. Standardized beta (95% CI) in basic and fully adjusted models; QC required ≥ 240 30-min bins. Fig. S5. Mean RA across AD-PRS quartiles. Means with 95% confidence intervals. Fig. S6. L5 and M10 timing across AD-PRS quartiles. Means with 95% confidence intervals. Fig. S7. Effect-size heatmap. Standardized beta values across outcomes, exposures, and covariate sets. Fig. S8. Lag sensitivity for AD-PRS. Estimates across 0-, 1-, 2-, and 5-year lag exclusions for incident dementia and incident AD (broad and strict). Fig. S9. Lag sensitivity for *APOE* ε4 dose. Estimates across 0-, 1-, 2-, and 5-year lag exclusions for incident dementia and incident AD (broad and strict). Fig. S10. Lag sensitivity for RA. Estimates across 0-, 1-, 2-, and 5-year lag exclusions for incident dementia and incident AD (broad and strict). Fig. S11. Endpoint definition sensitivity for Alzheimer’s disease. Broad (G30 or F00) versus strict (G30 only) definitions at lag = 0 for AD-PRS, *APOE* ε4 dose, and RA. Fig. S12. Competing-risk sensitivity for RA. Cause-specific Cox and Fine–Gray models with post-t0 counts for events of interest and competing deaths. Fig. S13. Overall-activity adjustment sensitivity for RA. Baseline fully adjusted model versus additional adjustment for mean acceleration (overall activity). Fig. S14. Selection-weighting sensitivity for RA. Unweighted versus inverse probability weighted (IPW) Cox models.

Additional file 2: Supplementary Tables S1–S9. Description: Multiple-testing results, genotype-category sensitivity analyses, ancestry restriction analyses, diagnostics, full Cox outputs, ICD-10 code lists, incident interaction analyses, mutual-adjustment sensitivity analyses, and the comparison of accelerometry-included versus excluded participants referenced in the main text. Table S1. Multiple-testing results for IS/IV. Raw *p* values with Bonferroni and BH-FDR corrections across 8 prespecified tests. Table S2. *APOE* genotype-category sensitivity analysis. Cross-sectional models comparing ε2/4, ε3/4, and ε4/4 groups with non-carriers, plus analyses excluding ε2/4. Table S3. European-only cross-sectional models. Regression outputs restricted to ancestry_group = 1. Table S4. IS/IV model diagnostics. Dropped covariates and diagnostics under QC ≥ 240 30-min bins. Table S5. Full Cox model outputs. Fully adjusted Cox outputs for incident dementia and incident Alzheimer’s disease. Table S6. ICD-10 code lists. Codes used for dementia and Alzheimer’s disease definitions (broad and strict). Table S7. Incident interaction analyses. Fully adjusted Cox models for RA × AD-PRS and RA × APOE ε4 dose in incident dementia and broad Alzheimer’s disease; all interaction *p* values are nominal. Table S8. Mutual-adjustment sensitivity analysis. Cross-sectional models including both AD-PRS and APOE ε4 dose for RA and phase-timing outcomes. Table S9. Accelerometry subcohort comparison. Baseline comparison of participants included in versus excluded from the accelerometry analytic cohort, used for the inverse-probability-weighted selection analysis.

Additional file 3 (optional): Analysis code (ZIP or repository link). Title: Analysis scripts for cohort assembly and statistical models. Description: Code sufficient to reproduce the main analyses, subject to UK Biobank terms of access.

## Data Availability

The datasets used and/or analysed during the current study are available from UK Biobank on reasonable request and subject to approval and terms of access (Application 1,162,548). Derived variables and analysis code are available from the corresponding authors on reasonable request, subject to UK Biobank terms.

## Abbreviations

AD: Alzheimer's disease
AD-PRS: Alzheimer's disease polygenic risk score
*APOE*: Apolipoprotein E
BH-FDR: Benjamini–Hochberg false discovery rate
BMI: Body mass index
CI: Confidence interval
HR: Hazard ratio
ICD-10: International Classification of Diseases, 10th revision
IS: Interdaily stability
IV: Intradaily variability
L5: Least active 5-h window
M10: Most active 10-h window
PC: Principal component
QC: Quality control
RA: Relative amplitude
RAP: Research Analysis Platform
UKB: UK Biobank
