# Supplementary material for: Alzheimer’s disease polygenic risk, APOE ε4 dose, and accelerometer-derived circadian rest–activity rhythms in relation to incident dementia and Alzheimer’s disease in UK Biobank: a prospective cohort study
Source: Alzheimers Res Ther. 2026 Jun 1;18:219. doi: 10.1186/s13195-026-02088-3 (PMC13628864; doi:10.1186/s13195-026-02088-3)

Figure S1. AD-PRS associations with relative amplitude and phase timing

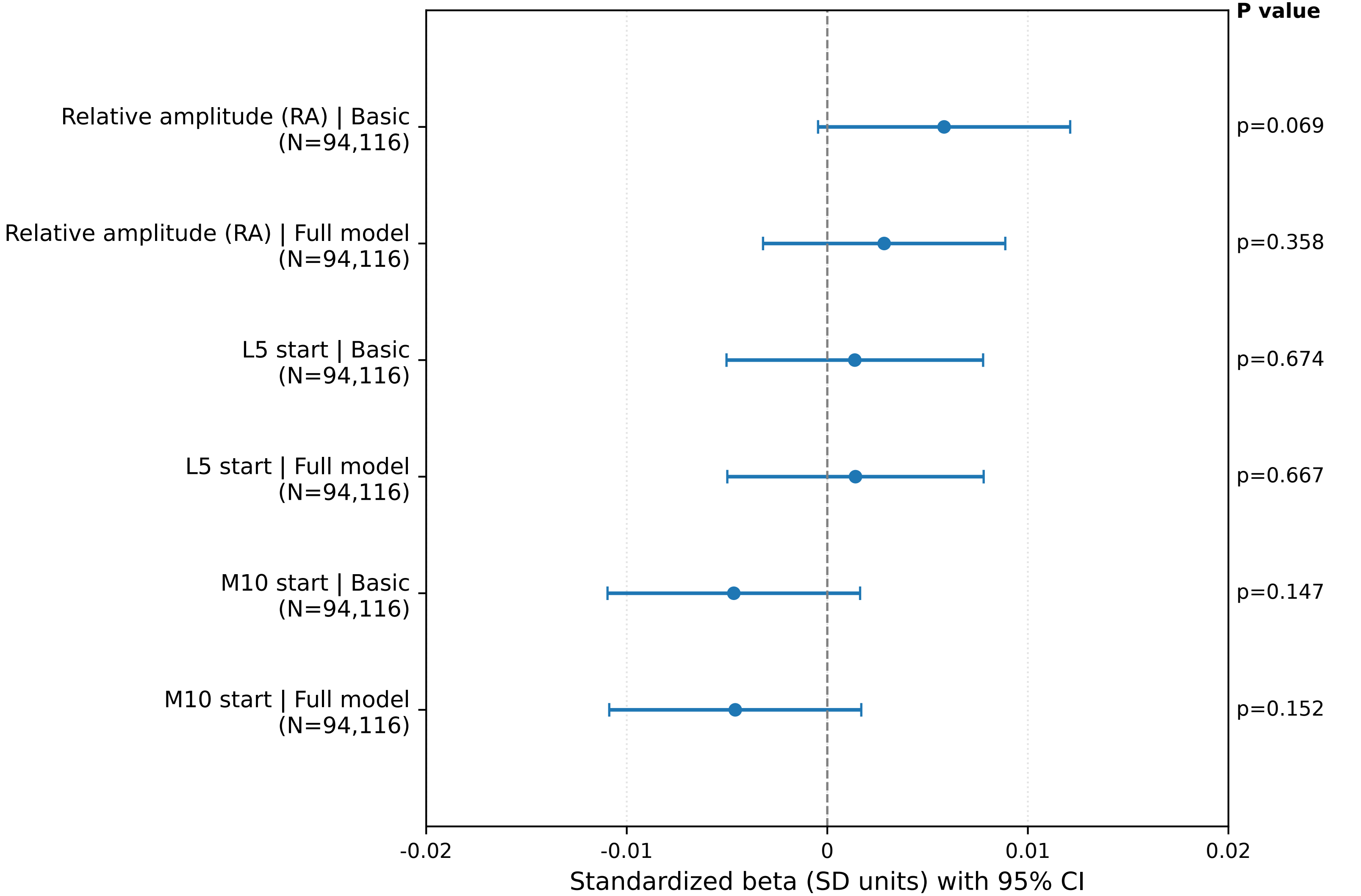

Figure S2. APOE ε4 dose associations with relative amplitude and phase timing

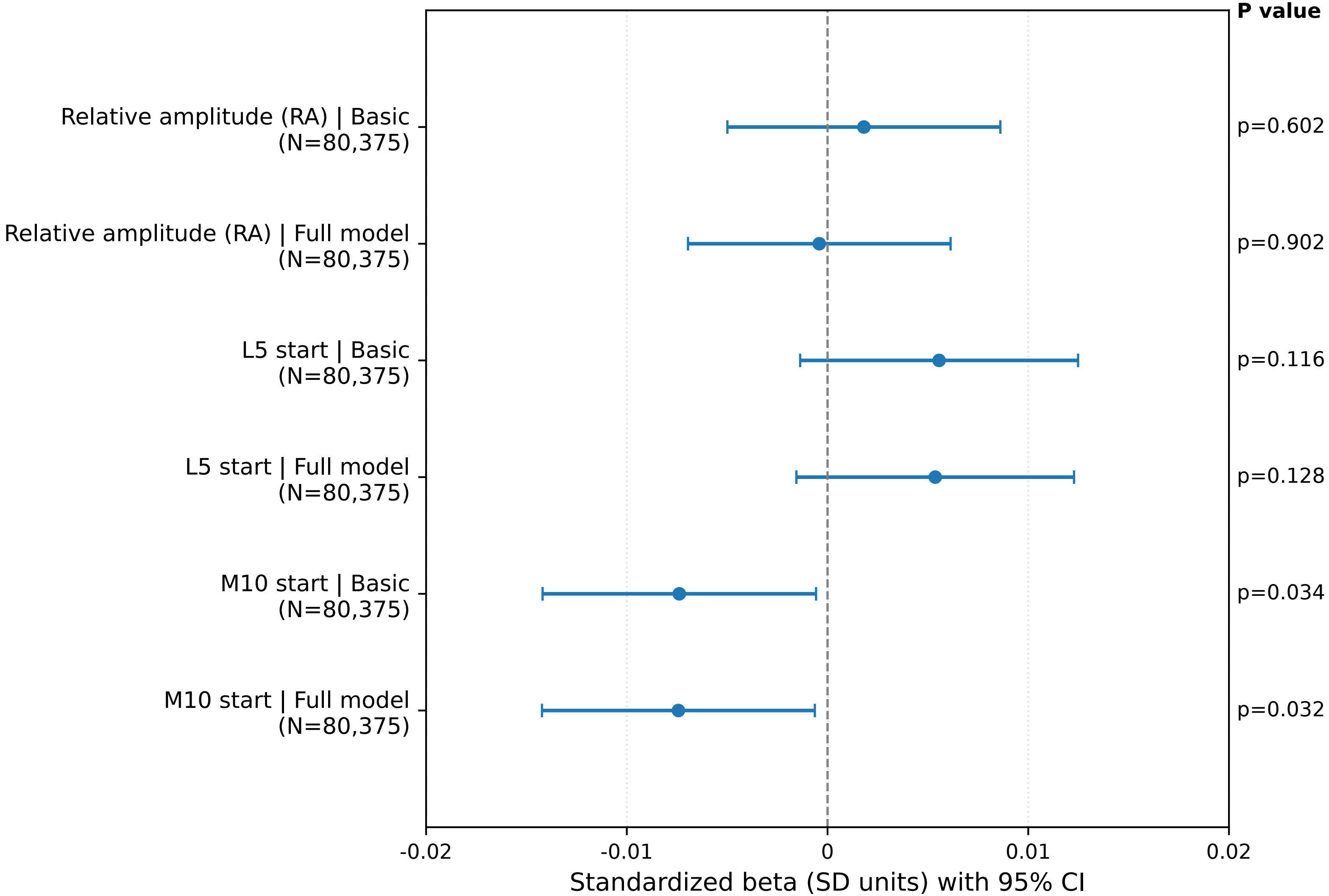

Figure S3. European-only sensitivity analysis for relative amplitude and phase timing

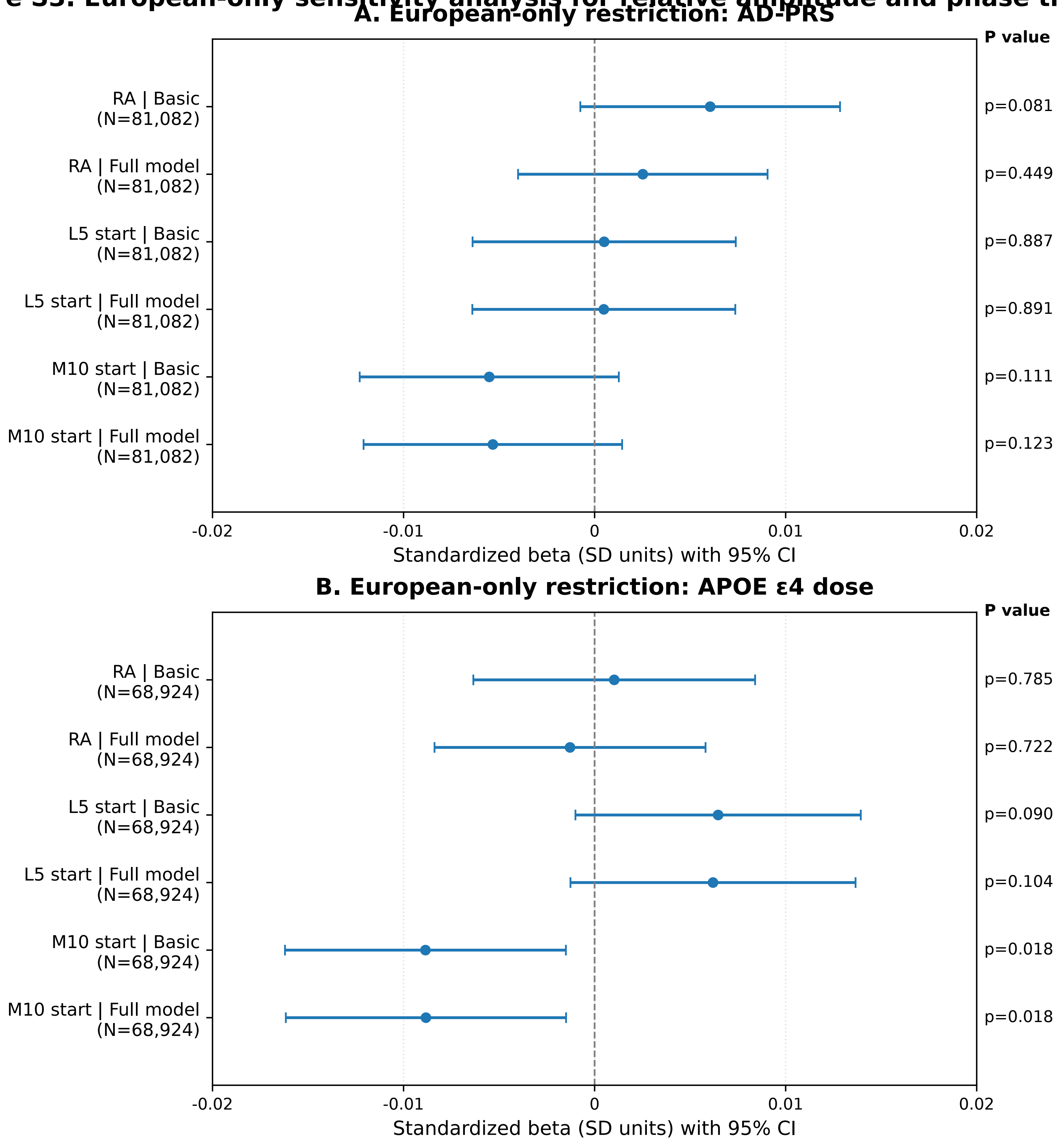

Figure S4A. AD-PRS associations with IS and IV

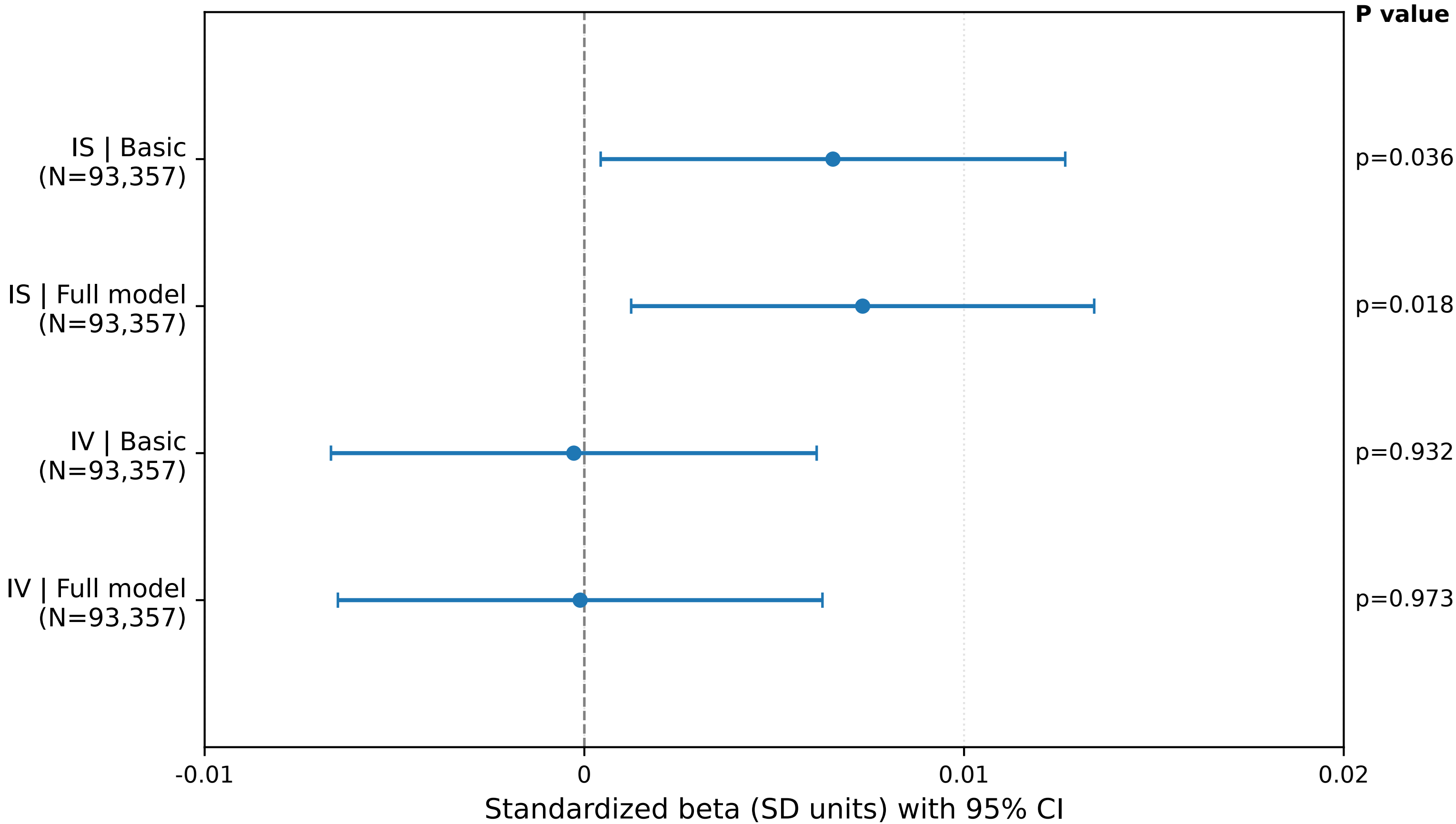

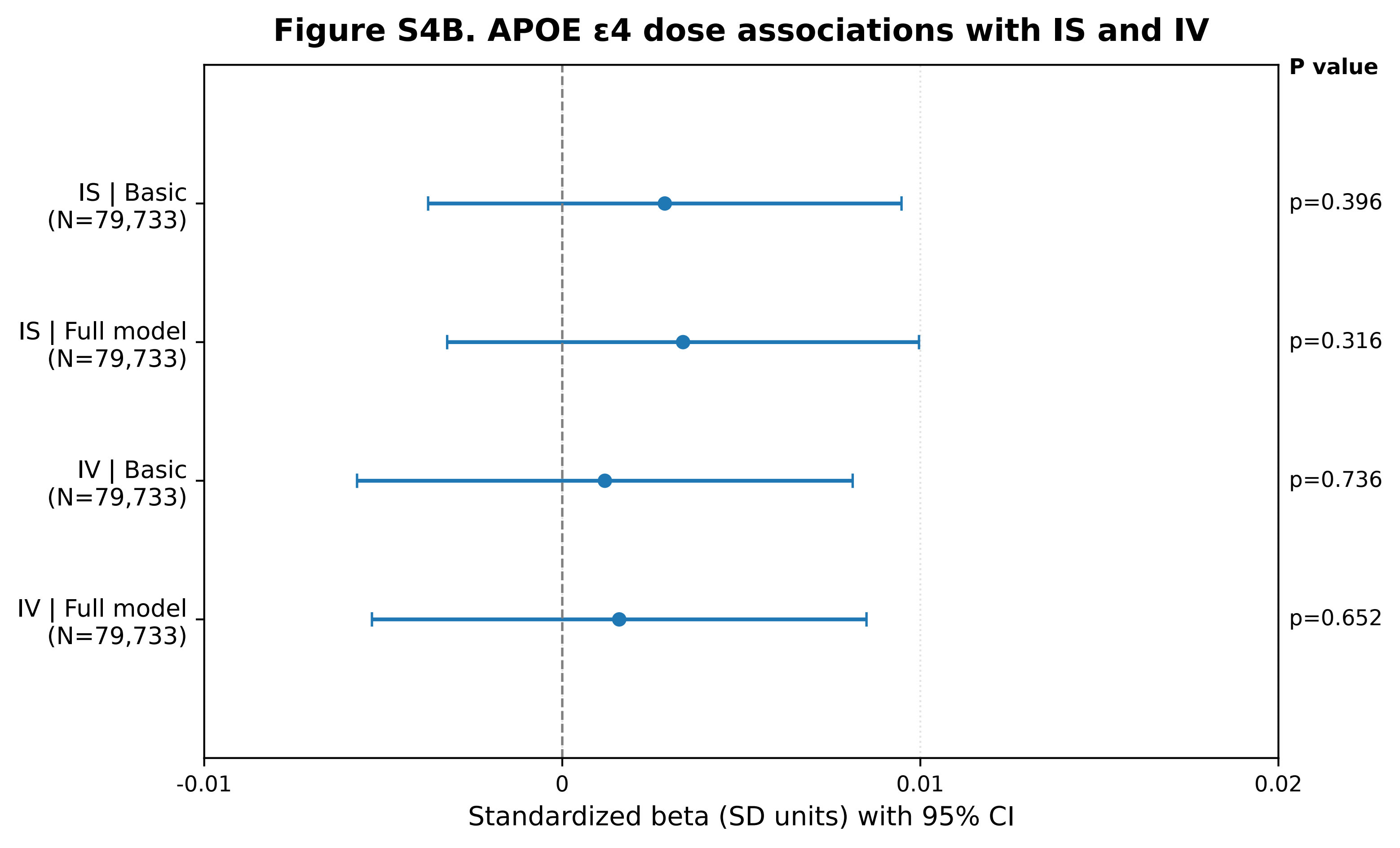
Figure S4B. APOE ε4 dose associations with IS and IV
P value
IS | Basic
(N=79,733)
p=0.396
IS | Full model
(N=79,733)
p=0.316
IV | Basic
(N=79,733)
p=0.736
IV | Full model
(N=79,733)
p=0.652
-0.01
0
0.01
0.02
Standardized beta (SD units) with 95% CI

Figure S5. Mean relative amplitude across AD-PRS quartiles

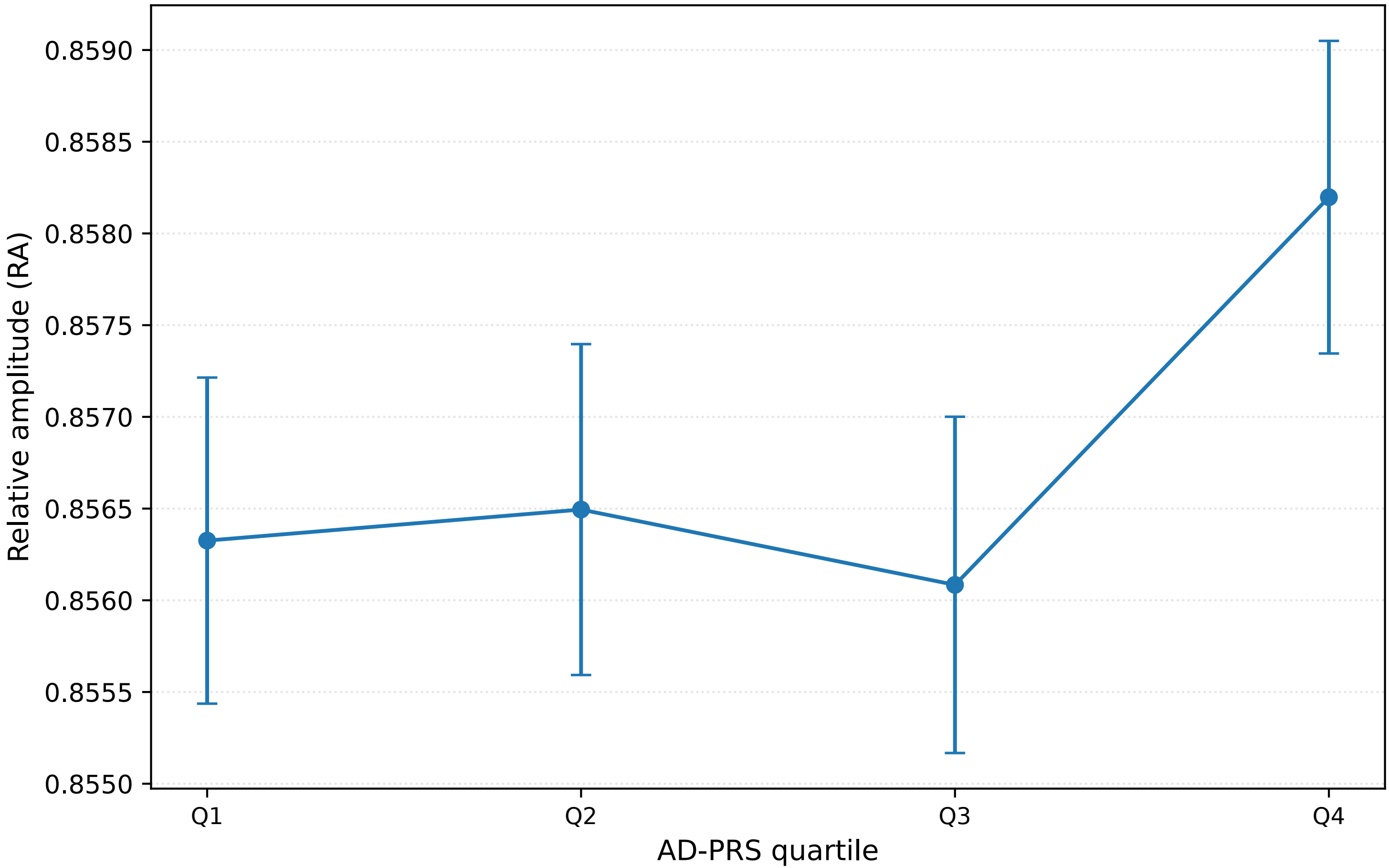

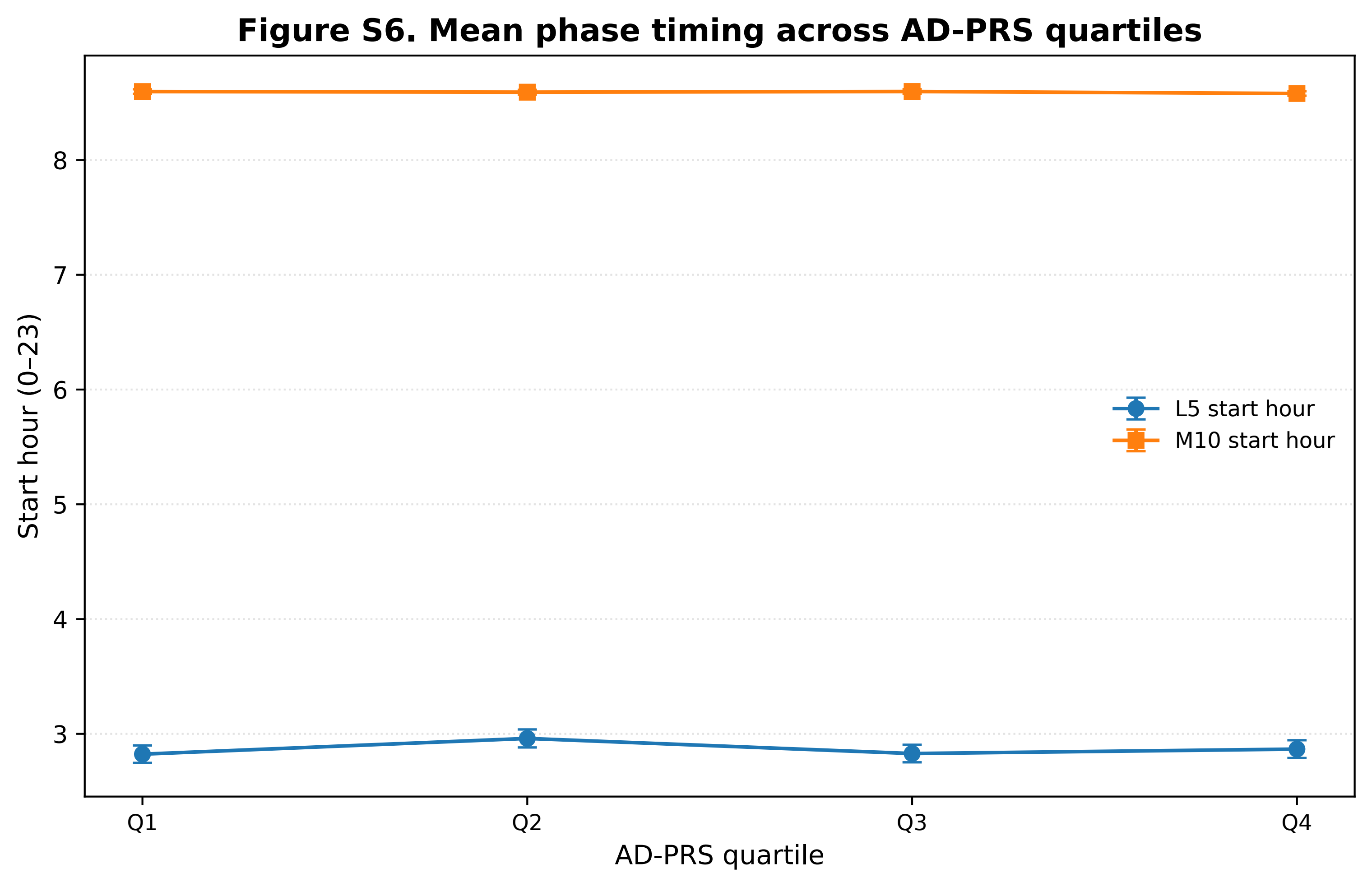

Figure S6. Mean phase timing across AD-PRS quartiles
Start hour (0–23)
3
4
5
6
7
8
Q1
Q2
Q3
Q4
AD-PRS quartile
L5 start hour
M10 start hour

Figure S7. Effect-size heatmap for cross-sectional results

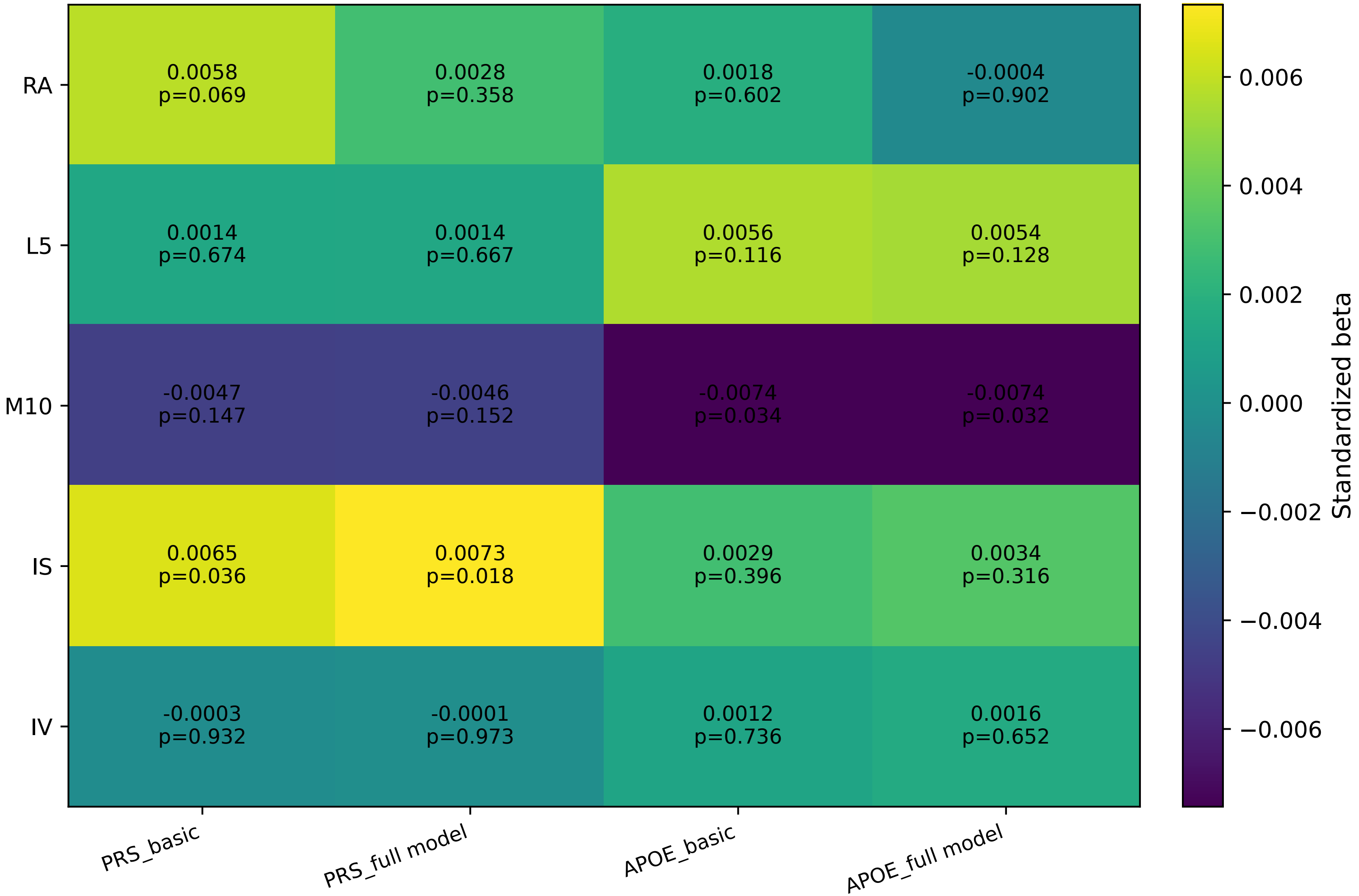

Figure S8. Lag sensitivity for AD-PRS (per SD)

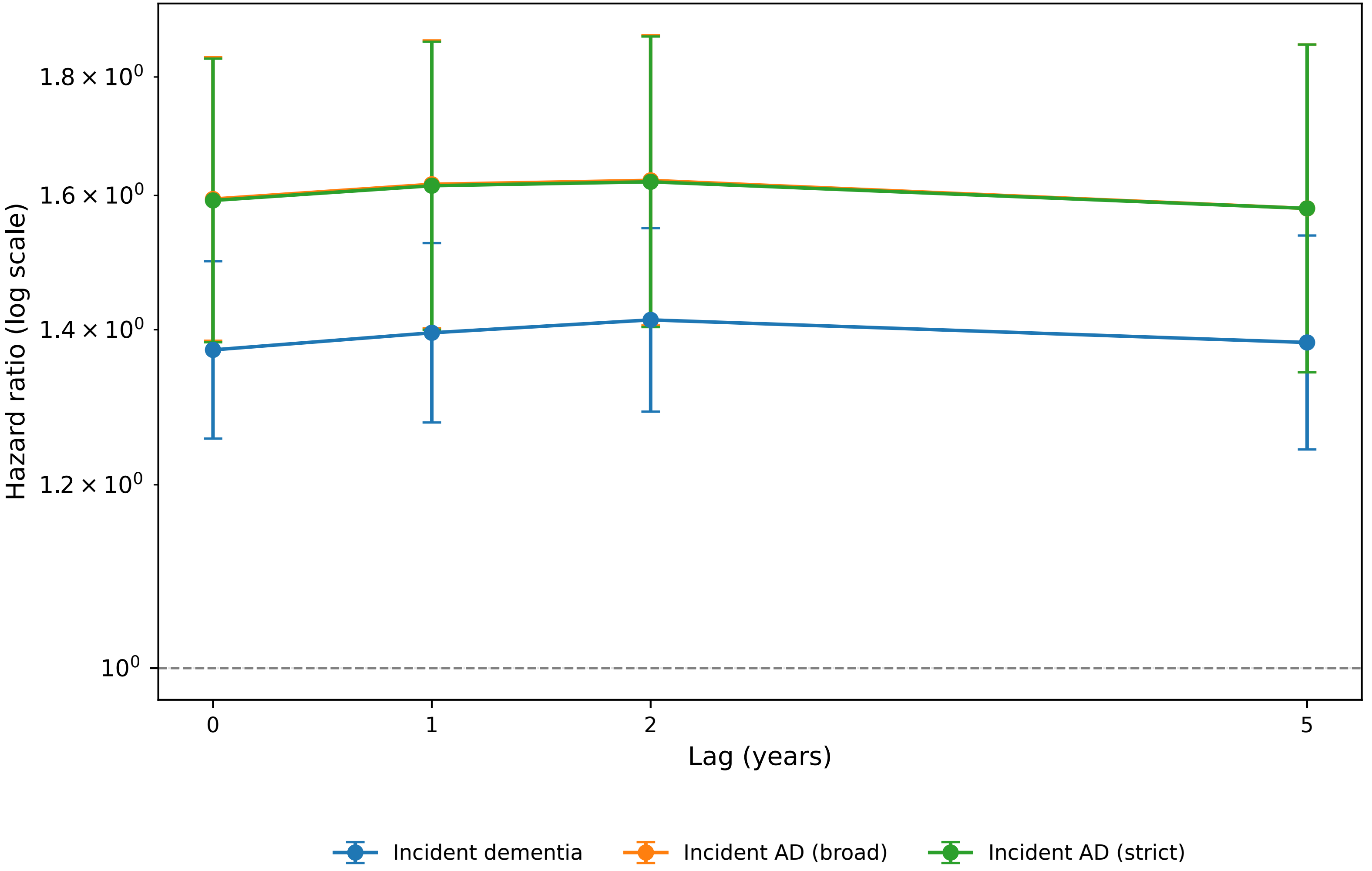

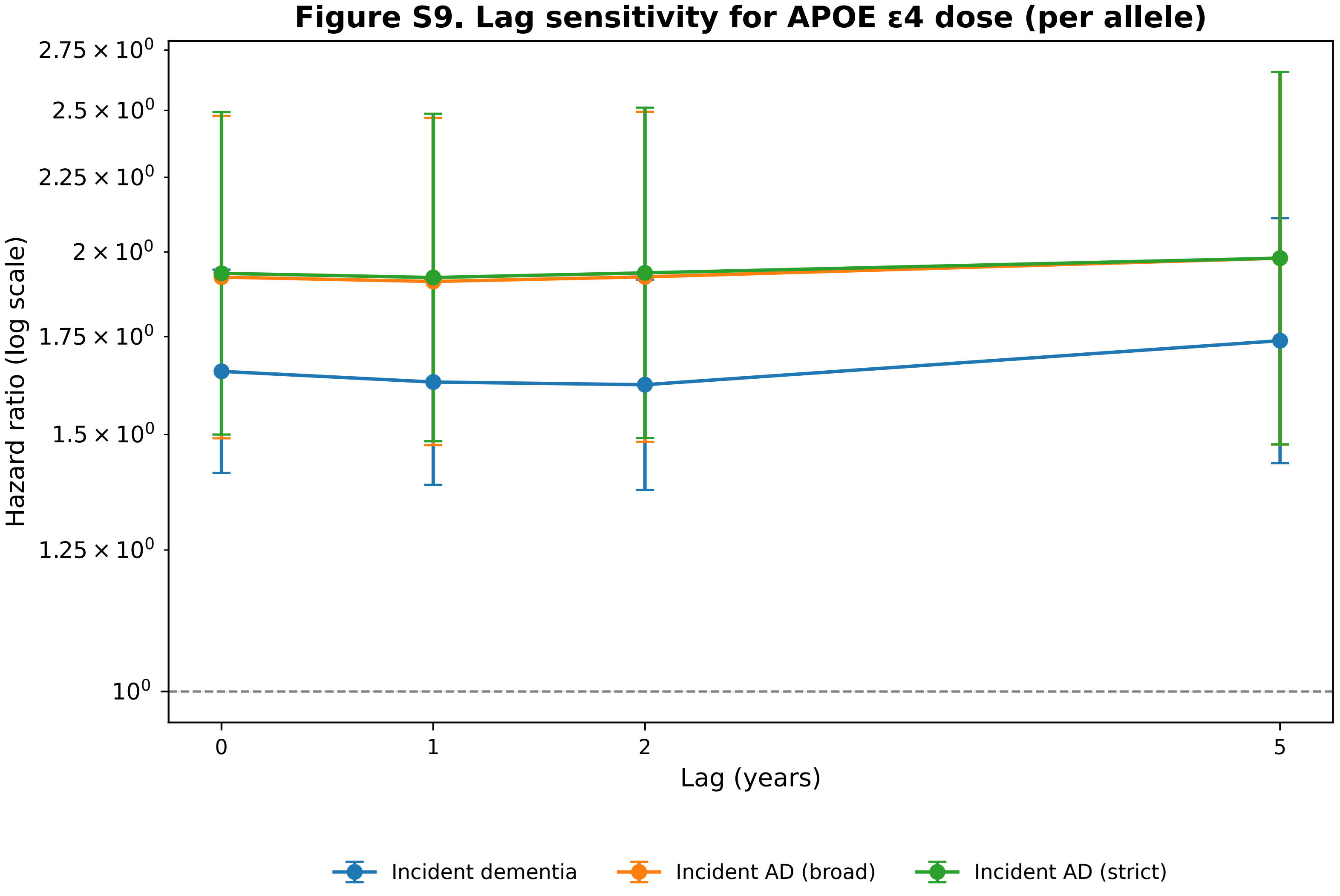

Figure S9. Lag sensitivity for APOE ε4 dose (per allele)
2.75 × 10^0
2.5 × 10^0
2.25 × 10^0
2 × 10^0
1.75 × 10^0
1.5 × 10^0
1.25 × 10^0
10^0
Hazard ratio (log scale)
0
1
2
5
Lag (years)
Incident dementia
Incident AD (broad)
Incident AD (strict)

Figure S10. Lag sensitivity for relative amplitude (RA; per SD)

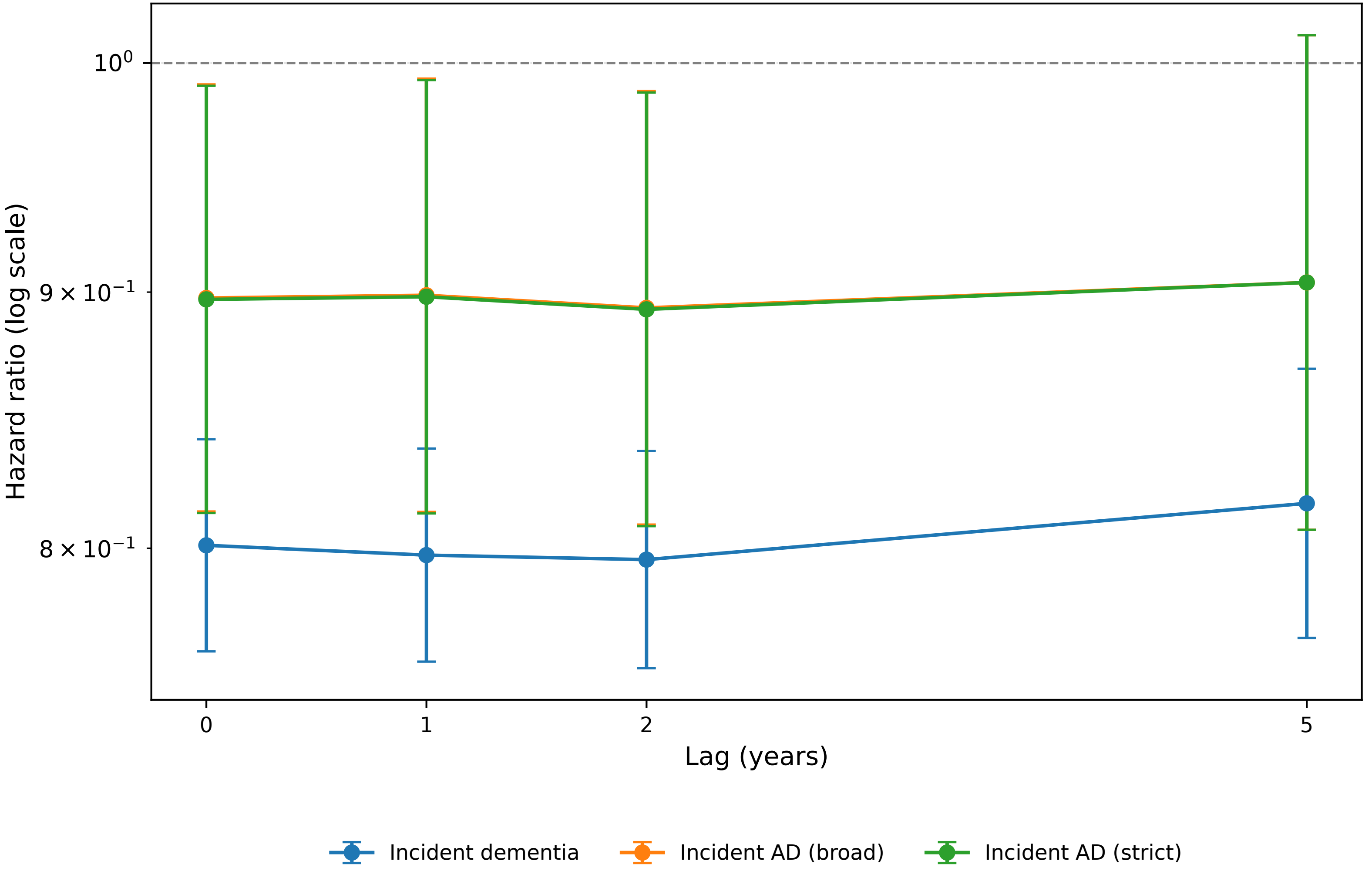

Figure S11. Endpoint sensitivity: broad vs strict Alzheimer's disease

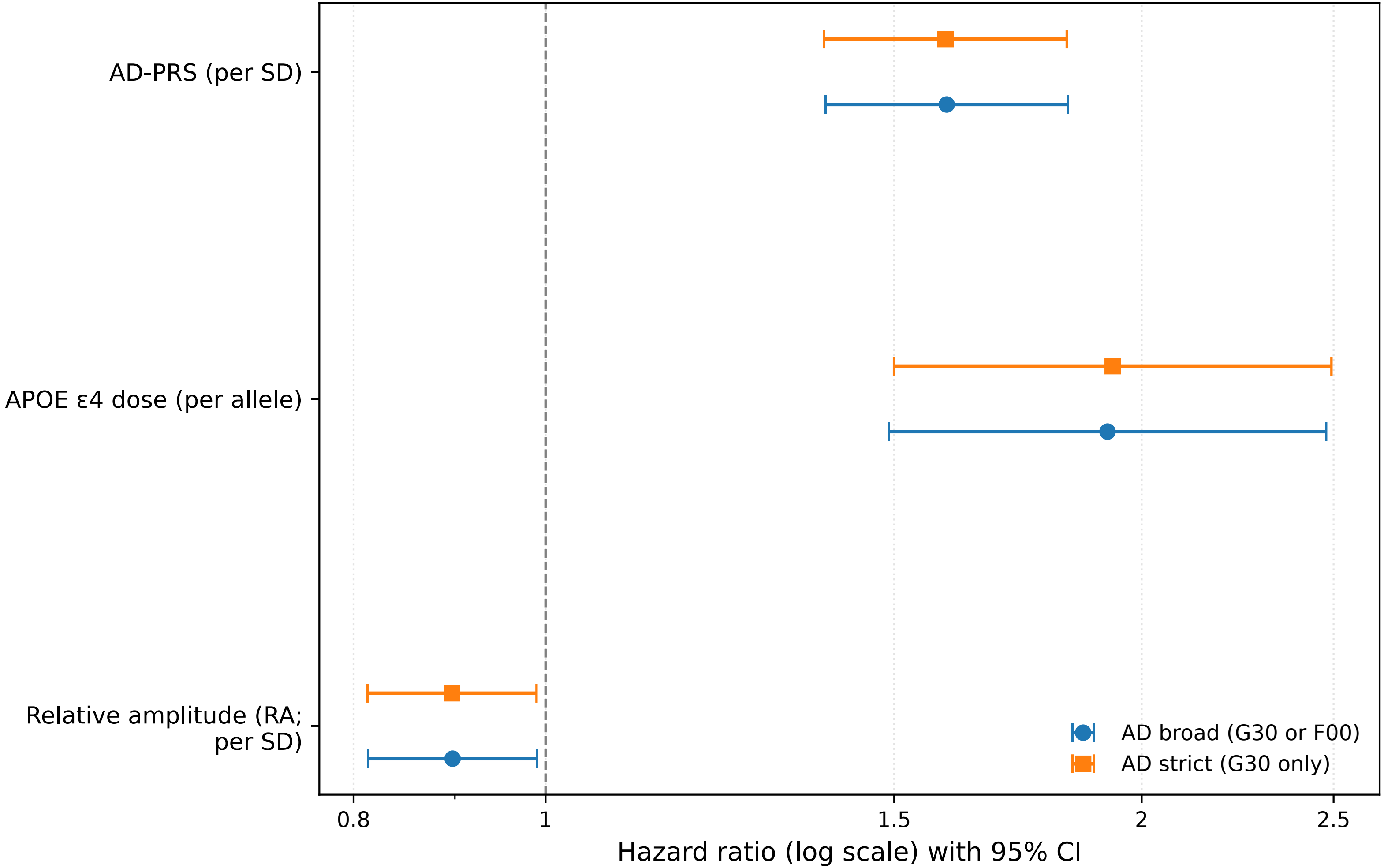

Figure S12A. Competing-risk sensitivity for relative amplitude (RA; per SD)

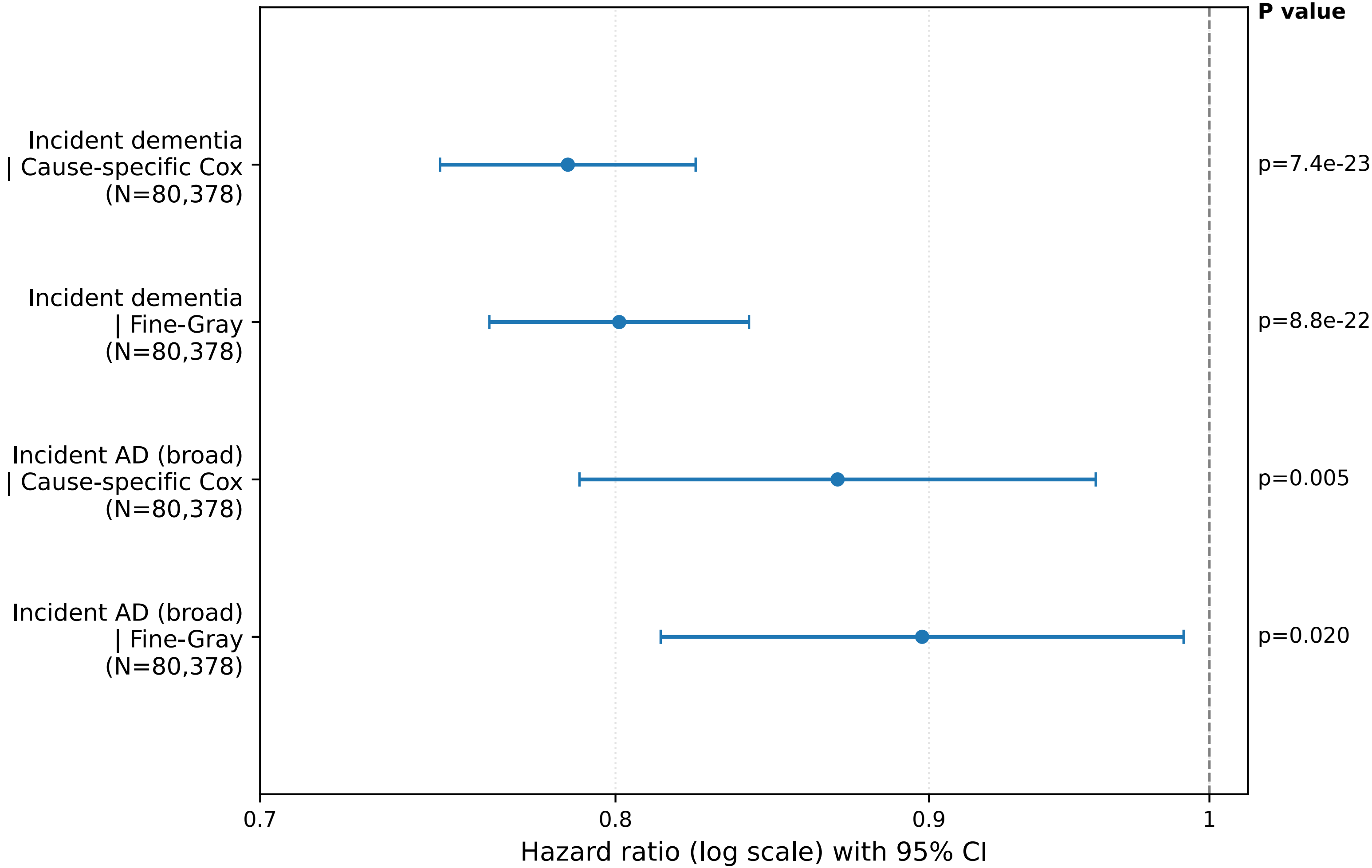

Figure S12B. Post-t0 event counts used in competing-risk analyses

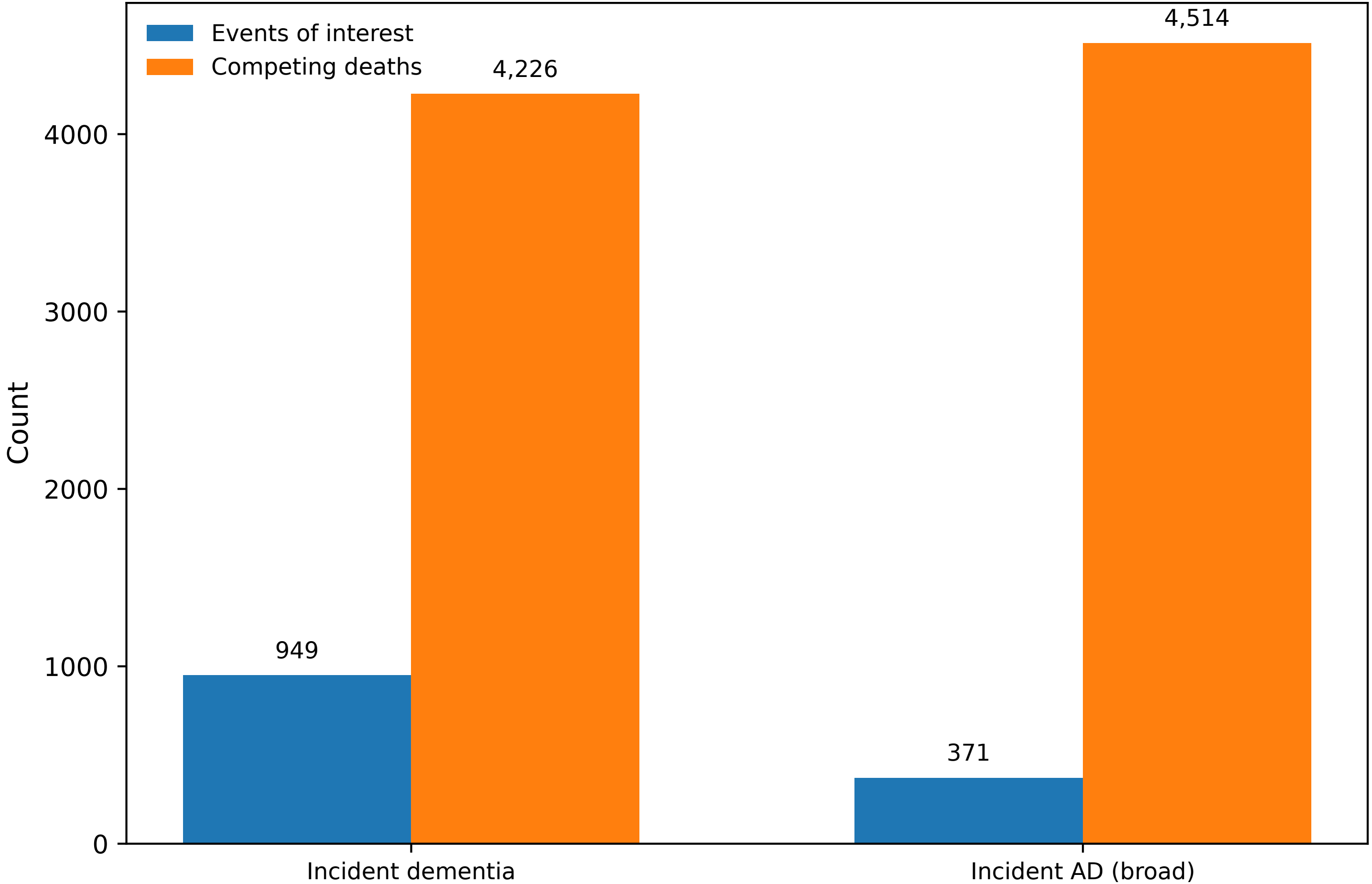

Figure S13. Additional adjustment for overall activity: relative amplitude

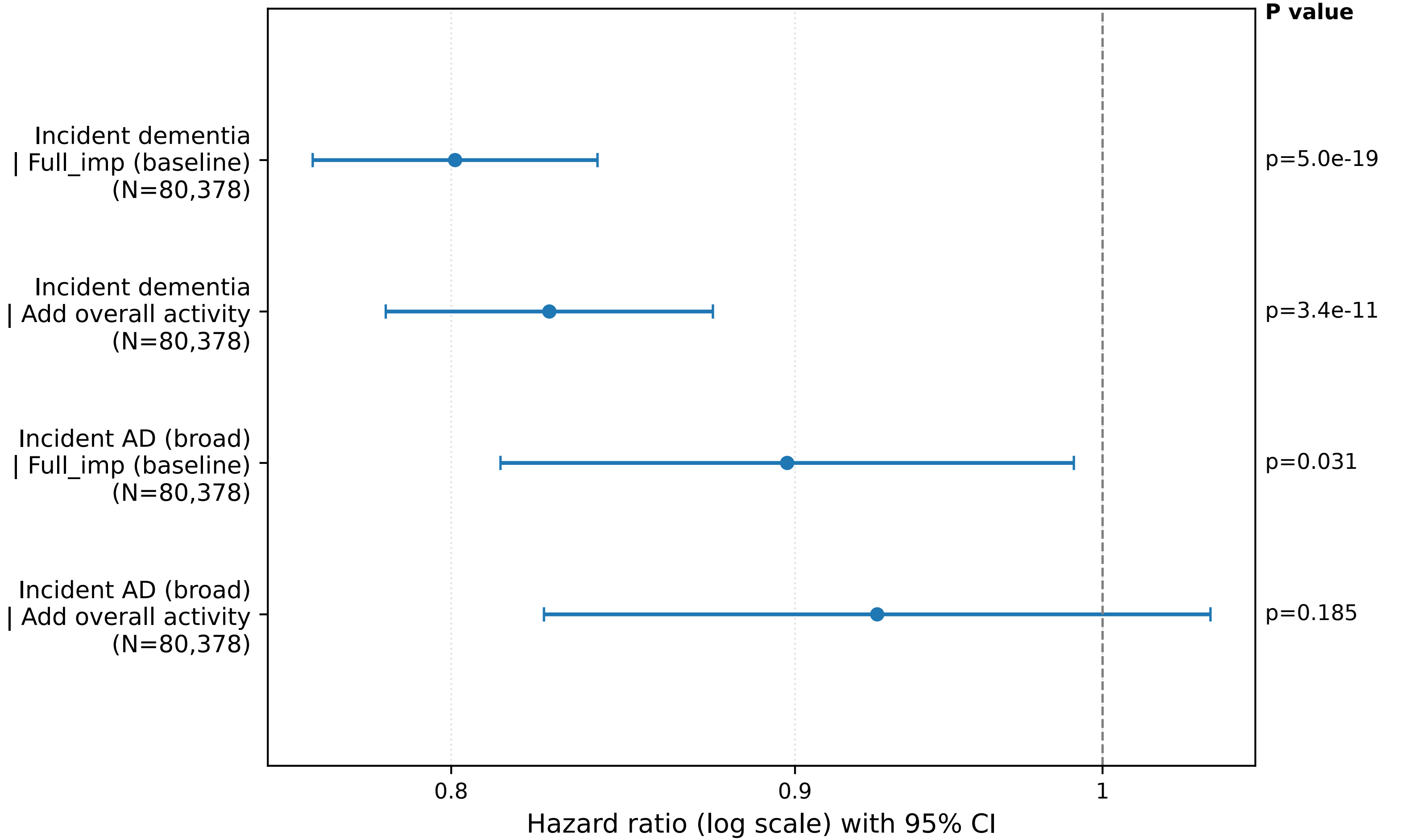

Figure S14. Inverse-probability-weighted sensitivity for relative amplitude

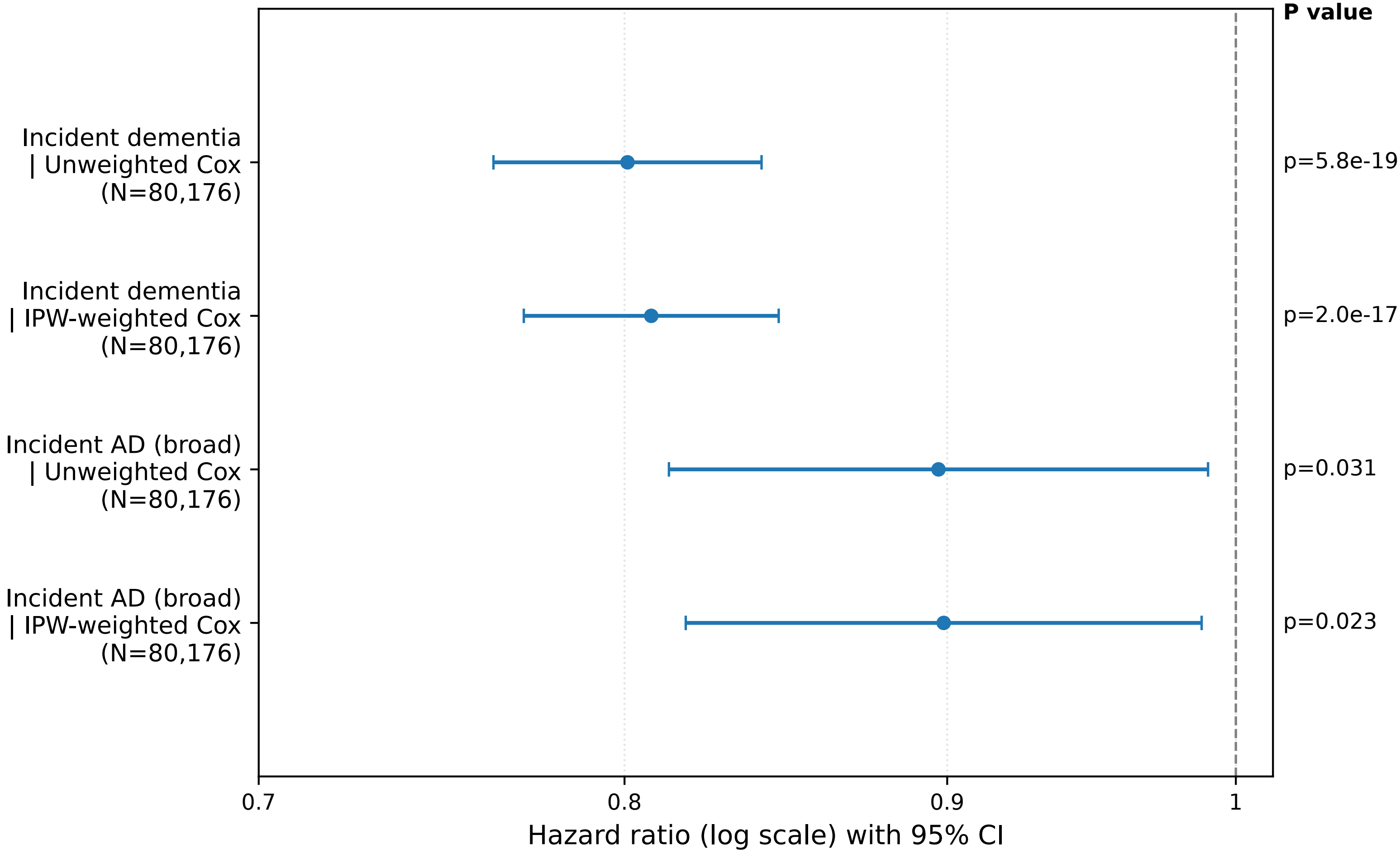

Supplement: Supplementary file 1 [file 13195_2026_2088_MOESM1_ESM.pdf]
